# Evaluation of a Model of Transitional Care After Preterm Birth on Parents’ Mental Health and Self-Efficacy: A Randomized Controlled Pilot Trial

**DOI:** 10.3390/children11101260

**Published:** 2024-10-18

**Authors:** Natascha Schuetz Haemmerli, Liliane Stoffel, Kai-Uwe Schmitt, Tilman Humpl, Mathias Nelle, Odile Stalder, Eva Cignacco

**Affiliations:** 1 Department of Health Professions, Bern University of Applied Sciences, 3008 Bern, Switzerland; kai-uwe.schmitt@bfh.ch (K.-U.S.); eva.cignacco@bfh.ch (E.C.); 2 Department of Paediatrics, Inselspital, Bern University Hospital, University of Bern, 3010 Bern, Switzerland; liliana.stoffel@bluewin.ch; 3 Graduate School for Health Sciences, University of Bern, 3008 Bern, Switzerland; 4 Insel Gruppe, Bern University Hospital, 3010 Bern, Switzerland; 5 Children’s Hospital Lörrach, 79539 Lörrach, Germany; humpl@icloud.com; 6 Departement of Children and Youths, Kantonsspital Baden, 5404 Baden, Switzerland; m.nelle@bluewin.ch; 7 Department of Clinical Research, University of Bern, 3008 Bern, Switzerland; odile.stalder@unibe.ch

**Keywords:** preterm infant, parents, transitional care, advanced practice, home visiting, randomized controlled pilot study, mental health

## Abstract

Background/Objectives: Parents of premature infants experience depression, anxiety, post-traumatic stress disorder, and increased stress, which can negatively impact parent–infant relationships and infant development. To reduce negative consequences and optimally support families, we developed the Transition to Home model (TtH). In this randomized controlled pilot trial (RCT), the feasibility of performing an experimental study to analyse the effects of TtH on parental mental health over time was evaluated. Methods: The following domains were assessed: recruitment, follow-up and study burden, outcome measures used and parental mental health outcomes. We included n = 22 parent couples with their preterm infants in the control group and n = 23 in the intervention group. Depression, anxiety and post-traumatic stress disorders, parenting stress, and parental self-efficacy were assessed at five timepoints. The study burden was evaluated once at the end of the study. Results: The control and intervention groups had similar socio-demographic characteristics. The groups showed no differences in the mental health outcomes except for depression in mothers at T2 (*p* = 0.042) and T5 (*p* = 0.027) and state anxiety in fathers at T2 (*p* = 0.016). Conclusions: This pilot RCT established a framework for the evaluation of the TtH model of care and demonstrated the viability of the evaluation scheme. The results confirm the suitability of the RCT’s structure and the feasibility of the methods and instruments used. Minor adjustments are recommended to include a more diverse sample in future studies.

## 1. Introduction

Over the past decades, remarkable advances in prenatal and neonatal care resulted in a higher survival rate for premature infants. This higher survival rate in turn leads to an elevated risk for neurodevelopmental and behavioral disorders [1,2]. Parents play an indispensable and crucial role in the care and surveillance of their premature infants, especially after hospital discharge [3]. However, a substantial number of these parents suffer from depression, anxiety, post-traumatic stress disorder, and increased stress, often coupled with a lack of self-confidence [4,5,6]. These emotional disturbances can lead to deteriorating parent–child relationships with short- and long-term negative effects on the infants. In contrast, preterm infants’ positive development and resilience are associated with mentally healthy parents who are sensitive toward their child [7]. Consequently, healthcare providers should be aware of the potential challenges and vulnerabilities faced by preterm children and their parents to provide support and to intervene at an early stage to prevent short- and long-term adverse effects in both, parents, and their preterm infants [8]. To accomplish this, various approaches are suggested.

The concept of family-centered care (FCC) is implemented in various healthcare areas, emphasizing the inclusion of family members to meet patients’ needs. In a neonatal intensive care unit (NICU), FCC interventions empower parents through collaboration, improve their understanding of caring for their preterm infant, address family-specific needs, and provide psychological support and family-centered information [9]. However, full integration of parents into the NICU care team remains limited in many programs [10]. To address this, the Family-Integrated Care (FICare) model was developed. The strength-based approach promotes parents as primary caregivers in the NICU empowers them, facilitates their learning, involves them in decision-making, and enhances self-efficacy, parent–infant relationships, and infant development post-discharge [11]. A cluster randomized controlled trial found that FICare reduces maternal stress and anxiety in parents of stable preterm infants [10,12], with similar results reported by Zhang et al. [13].

Various more specific care models have been developed to promote positive child development, eventually including an FCC approach. Most of these programs take place during the hospital stay and end with the child’s discharge. There are only a few programs that cover the entire care continuum from birth until the transition to home is fully completed. Examples of such programs include the Infant Behavioral Assessment Intervention Program (IBAIP) [14], the Stockholm Preterm Interaction-Based Intervention (SPIBI) [15], the transmural developmental support for very preterm infants and their parents (TOP-Program) [16], the modified Mother–Infant Transaction Program (MITP) [17], and the Transition to Home model (TtH) [18,19]. These intervention programs aim to support parents in understanding their child’s behavior and responding appropriately to their cues. They assume that infant behavior is influenced by interaction with the environment, including behavioral subsystems such as autonomy, motor skills, state organization, attention, and self-regulation [20].

Randomized clinical trials assessing care models have primarily concentrated on the child’s medical outcomes and development [14,17,21], with fewer studies exploring the psychological impact of care programs on parents [17,22]. Evaluating the IBAIP, no differences in total parenting stress after 12 and 24 months of corrected age were found [22]. A study examining the effectiveness of the MITP showed that mothers in the intervention group experienced less stress related to their child’s characteristics when the children were 3 months of corrected age. However, no significant differences were found in terms of maternal depression in mothers of children at 3 and 6 months of corrected age [17]. In Norway, a longitudinal RCT evaluated a modified version of the MITP including preterm children and their parents over a period from birth until the children were 9 years of age. At the corrected age of one and two years, mothers and fathers in the intervention group experienced significantly less stress compared to the preterm control group and the stress levels of the intervention group were comparable to those of the full-term control group [23]. While there is consensus that intervention programs for premature infants should follow a multi-level approach that includes FCC, psychosocial support, continuity of care, and successful interprofessional collaboration [24,25,26], it is challenging to evaluate such complex interventions.

The studies mentioned above illustrate this. The Medical Research Council (MRC) has thus published a framework for the development and evaluation process of complex interventions [27]. Four key stages were identified: (i) development, (ii) feasibility, (iii) evaluation, and (iv) implementation. This study will focus on the feasibility, i.e., the second stage, according to aspects of MRC such as the feasibility of the recruitment and retention of participants, adequate sample size, the feasibility of data collection (e.g., appropriate instruments), and time and personnel resources for data collection and analysis, and will also address unforeseen outcomes that are relevant.

### Aims

This randomized controlled pilot trial aimed at exploring a strategy to assess the acceptability and feasibility of the Transition to Home (TtH) model of care that was developed in Switzerland [18,19]. It was the intention to provide methods and procedures needed to conduct a full-scale randomized controlled trial. In this first approach, an evaluation concept was developed and implemented to check for the effects of the intervention on parents’ depression, anxiety, post-traumatic stress disorders, parenting stress, and self-efficacy over time until six months after hospital discharge.

## 2. Materials and Methods

### 2.1. Study Design

We conducted a non-blinded pilot randomized controlled trial with a two-arm design over time. The reporting of this trial follows the CONSORT guidelines [28] with its extension to randomized pilot and feasibility trials [29].

The trial was registered at ClinicalTrials.gov on 23 January 2018 under NCT03460496.

The study and amendments were approved by the responsible ethics committee of the canton of Bern, Switzerland (Project-ID: 2017-01249).

### 2.2. Setting and Participants

The pilot trial was conducted at the neonatal intensive care unit (NICU) of the Department of Pediatrics, Inselspital, University Hospital, Bern, Switzerland between February 2018 and March 2020. The NICU is a tertiary referral center with 700 to 800 neonates annually. Parents with their preterm infants admitted to the NICU of the University Hospital were eligible if (a) preterm infants were born between 24 0/7 and 34 6/7 weeks of GA; (b) they were hospitalized at the University Hospital; (c) they resided in the canton of Bern (corresponds to a state); (d) they were fluent in spoken and written German, French or English. We excluded preterm infants with congenital problems evident at birth.

A team of 18 trained nurses conducted the eligibility assessment and recruited parents and children at the earliest 48 h after birth and as soon as the mother and child were in a stable health situation. A nurse in the recruiting team informed parents in detail about the study and asked them to participate. If necessary, parents were offered further discussions. After the written informed consent of parents and their infants was obtained, families were randomly assigned to the intervention (IG) or control group (CG). The first (N.S.H.) or second author (L.S.) performed randomization after they were informed about participation through the recruiting nurse. Families whose children were transferred to another hospital during the recruitment process could not participate in the study. When participants of the IG were transferred to another hospital after randomization, they were considered lost to follow-up because we were not able to provide the intervention.

### 2.3. Sample Size

We aimed to include a convenience sample of 46 families, with 23 families in each group. The sample size was estimated based on the number of patients of previous years assuming a recruitment duration of 6 months. Given the pilot nature of this study, a power analysis was not conducted. However, the results can be used to do so.

### 2.4. Randomization

A computer-generated probabilistic minimization was used to randomize families to either the CG or the IG, accounting for gestational age (GA) in weeks in three groups: i. 24 0/7 to 27 6/7 weeks; ii. 28 0/7 to 31 6/7 weeks; and iii: 32 0/7 to 31 6/7 weeks. We further accounted for multiple versus single births.

### 2.5. Standard Care

Infants and parents allocated to the CG received standard care at the study site. This usual care in the NICU includes concepts such as kangaroo care [30], development-enhancing care (e.g., basal stimulation, kinaesthetic infant handling) [31,32], primary nursing [33], the program “Creating Opportunities for Parent Empowerment (COPE)” [34], nursing educational sessions for nutrition and sleep, nonstop visiting hours, and regular medical briefings. During hospital stay services such as psychological support, social support, lactation consultations and music therapy were offered on request, but not as a standardised service. After hospital discharge, participants of the CG were supported on their own initiative by a midwife, the paediatrician, the family advisory service or other support services.

### 2.6. The “Transition to Home (TtH)” Model in Addition to Standard Care

The “Transition to Home (TtH)” model was designed to optimize transitional care of families with preterm infants. Focus is set on the transition from hospital to home, and the model considers the entire family [18,19]. TtH aims to give parents and infants structured, individual support in order to improve parents’ mental health and competence to promote the child’s development and optimize interprofessional collaboration. 

The TtH model is based on a German model that supports families with chronically and critically ill children [35] and on Naylor’s “transitional care model”, a model developed in the United States for hospitalized elders [36]. Naylor’s model is based on the model developed by Brooten et al. [37] in which a specialized nurse with advanced knowledge in neonatology prepares families with very low birth weight infants for earlier discharge. The perspectives of users and healthcare professionals were considered in the development of the TtH model.

#### 2.6.1. Advanced-Practice Nurse Support

The core component of the TtH model is the continuous support of the family provided by an Advanced-Practice Nurse (APN). The APN is an academically trained and neonatology-specialized nurse. Advanced practice nursing competencies include, among others, clinical practice, counselling, support of other healthcare professionals and leadership tasks [38,39]. While APNs are internationally established, they are still emerging in Switzerland such that few role models are available in the context of this healthcare system [40,41]. In the TtH model, the APN headed a team of three experienced nurses in the NICU. 

A member of the Advanced Nursing Practice (ANP) team is the primary contact person for the family from birth, during hospitalization and until six months after discharge. An ANP team member is available during the day shift four days (Monday to Thursday) per week. The ANP team member coordinates the interventions of the interprofessional team during the whole support period. This includes a contribution to a comprehensive plan for individual discharges, holding consultations, and close collaboration with different healthcare professionals and participation in regular interprofessional exchanges. The APN leads interprofessional roundtable discussions with involved healthcare professionals (HCP) and parents; these discussions were newly established as part of the model and are held twice while the preterm infant is hospitalized and once three months after discharge. The meetings seek consensus on optimal support for families in care.

The APN’s interventions focus on strengthening parents’ competencies, their self-efficacy and promoting a positive parent–infant interaction. Therefore, the APN takes a family-centered approach in assessing the needs of the families and in making shared decisions. The APN further provides the “Newborn Behavioral Observation” (NBO), an infant-centered and family-focused method for building relationships, to parents and children [42] during and after hospitalization. The NBO helps sensitize parents to their infant’s competencies and capabilities, teaching them to read their infant’s signals and understand their behaviors. The APN regularly visits consults with, and educates parents and acts as a continuous partner during hospitalization and after discharge. 

After discharge, the APN offers systematic follow-up calls, telephone support and home visits to assess the physical health of infants and parents and the mental health of parents, with a view to evaluating interventions and adapting the care as the family’s needs evolve. Within the first 24 h after the preterm infant’s discharge, the APN conducts a first follow-up telephone consultation. Two further phone calls take place approximately on day 21 and day 52. On day 3 after discharge, a first home visit takes place, followed by additional home visits approximately every 15 to 30 days. The timing and number of the follow-up calls and visits are adapted according to each family’s individual needs. Toward the end of the period, the APN determines, with the parents and other HCP, if the family needs further support, and which specialist (e.g., an early childhood healthcare worker) would be most appropriate to continue the collaboration with the family.

#### 2.6.2. Enhanced Support from Different Healthcare Professionals

In addition to the APN support, the families in the IG receive specific support from different healthcare professionals during their hospital stay and if needed after discharge. The difference from the CG is that families receive support in a structured way, and it is guaranteed in any case. 

A psychologist provides psychological support to all families, comprising assessment and at least three follow-up consultations before the infant is discharged. The goal is to re-establish emotional stability, improve parents’ ability to cope with the situation, prevent parents and family from developing adaptive disorders, and protect the infant from developmental disorders.

A lactation consultant provides as many consultations as the family needs to reach the competencies to meet their child’s nutritional needs and strengthen parent–child bonds.

The physical therapist provides treatment after an assessment as soon as the preterm infant is in a stable health condition. In a single consultation, the family learns how to handle their premature infant in everyday life in a manner appropriate to the infant’s developmental stage.

### 2.7. Date Collection and Outcomes

We collected data through self-report questionnaires, which were completed by mothers and fathers independently at 5 different timepoints: after birth and group allocation = baseline (T1); at 35 weeks of gestational age and before discharge (T2); one week after discharge from hospital (T3); three months after discharge (T4); and six months after discharge (T5). 

After the randomized controlled group allocation, the recruiting nurse provided the baseline questionnaire (T1) to the participating parents. Parents in the IG were contacted by their assigned APN and were introduced to the intervention. The questionnaires to collect data at T2, T3, T4 and T5 were sent to parents of both groups, IG and CG, by mail with a prepaid reply envelope. If the questionnaires were not returned within two weeks, the parents received a written reminder and a telephone reminder after an additional three weeks.

#### 2.7.1. Sociodemographic Data

Sociodemographic data were self-reported via baseline (T1) questionnaires and included parents’ age, gender, nationality, mother tongue, living situation, marital status, educational level, occupational situation, yearly family income and the preterm infants’ gender, gestational age at birth, birth weight, birth length and length of hospital stay. We further assessed the mode of delivery, pregnancy diseases, multiple births and the total number of children per family.

#### 2.7.2. Feasibility Measures

Study burden was measured at the end of the study period at T5 with a Visual Analog Scale (VAS) [43]. Parents marked a spot along a line that represents a continuum of burden related to their participation (range from 0 = low to 10 = high).

#### 2.7.3. Mental Health Measures

We assessed parental mental health in terms of depression, anxiety and post-traumatic stress disorders, parenting stress and parental self-efficacy at all five timepoints.

##### Depression

The Center for Epidemiological Studies Depression Scale (CES-D) includes twenty items comprising six scales reflecting major facets of depression [44]: depressed mood, feelings of guilt and worthlessness, feelings of helplessness and hopelessness, psychomotor retardation, loss of appetite, and sleep disturbance. For each item, respondents rate the frequency of their symptoms over the past week on a 4-point scale ranging from 0 to 3. All items’ scores are summed to yield a total score, ranging from 0 to 60. Higher scores indicate more depressive symptoms, with a score of 16 or higher identified as the clinical cut-off score for depressive symptoms.

##### Anxiety

The State-Trait Anxiety Inventory (STAI) assesses parental anxiety and asks 20 questions to measure State Anxiety (State AI), and 20 questions for Trait Anxiety (Trait AI) [45]. Responses are scored on 4-point forced-choice Likert-type scales ranging from 20 to 80. A higher score corresponds to greater anxiety. A cut-off score of 39–40 has been suggested to detect clinically significant symptoms of state anxiety.

##### Post-Traumatic Stress Disorder (PTSD)

The PCL-5 checklist assesses symptoms of PTSD [46]. It contains 20 items that are rated on a 5-point Likert-type scale from 0 (not at all) to 4 (extremely). The items refer to the past month pertaining to a specific event that caused the trauma. Total scores range from 0 to 80 and a cut-off score of 31 to 33 is recommended indicating PTSD being present.

##### Parenting Stress

The Parenting Stress Index Short Form (PSI–SF) is designed to measure stress in the parent–child system [47]. The PSI–SF consists of three subscales: parental distress (PD), parent–child dysfunctional interaction (PC dys. int.), and difficult child (Diff. Ch.). Each subscale consists of 12 items rated from 1 (strongly disagree) to 5 (strongly agree). Subscale scores therefore range from 12 to 60, whereas the total score ranges from 36 to 180. High scores on the subscales and PSI–SF total score indicate greater levels of stress. The parental distress subscale reflects a parent’s perception of child-rearing competence, conflict with his or her spouse or partner, social support, and stresses associated with the restrictions placed on other life roles. The parent–child dysfunctional interaction subscale assesses a parent’s perception that the child does not meet expectations and that interactions with the child are not reinforcing. The difficult child subscale surveys the parent’s view of the child’s temperament, defiance, noncompliance, and demandingness.

##### Parental Self-Efficacy

The Tool to measure Parenting Self-Efficacy (TOPSE) is an instrument of 48 statements that encompasses eight dimensions of parenting [48]: emotion and affection; play and enjoyment; empathy and understanding; control; discipline and boundaries; pressures; self-acceptance; and, learning and knowledge. The items are rated on an 11-point Likert scale and then summed to obtain the final score. Lower scores reflect lower levels of parenting self-efficacy. The German version of the TOPSE, translated and adapted by Hirter et al. [49], was reduced to 30 items that encompass five dimensions of parenting (emotion and affection, empathy and understanding, pressures, self-acceptance and learning and knowledge). This reduction was made because the dimensions “play and enjoyment”, “control”, and “discipline and setting boundary” are not applicable to postpartum parents. The adaptation of the instrument was conducted in consultation with the developers of the instrument [49].

### 2.8. Statistical Analysis

Study data were entered and managed using Secu Trial, a secure electronic research data capture system hosted at the University of Bern. All analyses were carried out using Stata version 17.0 [50].

We compared the demographic characteristics of the study participants with non-participants separately for mothers and fathers or other caregivers using the Chi-squared test and the non-parametric Wilcoxon rank-sum test to compare categorical variables and continuous variables, respectively.

The number of dropouts and their timepoints were recorded and displayed separately for mothers and fathers. Missing items in the different scores were imputed. If there were less than 20% of missing items in a score or sub-score, the missing items were replaced by the mean of the other available items from the same score or sub-score. Following the PSI-SF score’s guidelines [47], only one missing item per sub-score was allowed for the imputation by the mean of the other items from the same sub-score.

We compared baseline characteristics of participants by arm (Chi-squared test or Wilcoxon test). Median, interquartile range (IQ-range), mean and standard deviation (SD) for each score were computed by visit and by parents. Numbers and proportions of high scores were computed for the binary scores by parent and visit timepoint. We computed the proportions of babies’ characteristics by visit and groups. Median, IQ-range, mean and SD of babies’ corrected age at the different visits (T1, T2, T3, T4 and T5) were computed by arm and compared using the non-parametric Wilcoxon rank-sum test and t-test as appropriate. The median and mean of corrected age at discharge were computed and compared between both arms. 

For continuous outcomes, the adjusted mean difference between the intervention group (IG) and control group (CG) and its 95%-CI were computed through repeated-measures mixed-effects linear regression models. Adjustment was carried out for stratification factors used in randomization (multiple births and preterm group) with a random intercept for subjects to account for the correlation of outcome data between multiple timepoints. An interaction term between the randomization group and time was also considered. If baseline data were available (CES-D, STAI, PTSD, PSI-SF and TOPSE), the model was adjusted for the baseline value. Crude means and their 95%-CI were also computed by group and timepoint. The adjusted mean difference for study burden was computed with ordinary linear regression because it was only measured at one timepoint (T5, 6 months after discharge). Mothers and fathers were analysed separately. The woman as the other caregiver was considered with mothers. For this family with two mothers, cluster-robust standard errors for families were included. A negative difference represents a lower score in the IGs compared to the CGs and vice versa. These model-based analyses were carried out based on the Full Analysis Set (FAS) of participants. Scores’ means and their 95%-CI were computed and represented in graphs by visit and randomization group separately for mothers and fathers.

Secondary analyses were carried out based on the per-protocol set (PPS) of participants using the same statistical models and methods as the main analysis. Intra-class correlation (ICC) for mothers and for fathers for all scores between all timepoints was also computed, and pairwise correlation, with all timepoints combined, was computed between fathers and mothers for all scores.

The effect size was computed through Cohen’s d (difference between two means divided by a standard deviation for the data). These effect sizes were computed for both sets of data (FAS and PPS) separately for mothers and fathers for all the different outcomes at all visits.

## 3. Results

### 3.1. Feasibility

#### 3.1.1. Recruitment Process

**Figure 1:**
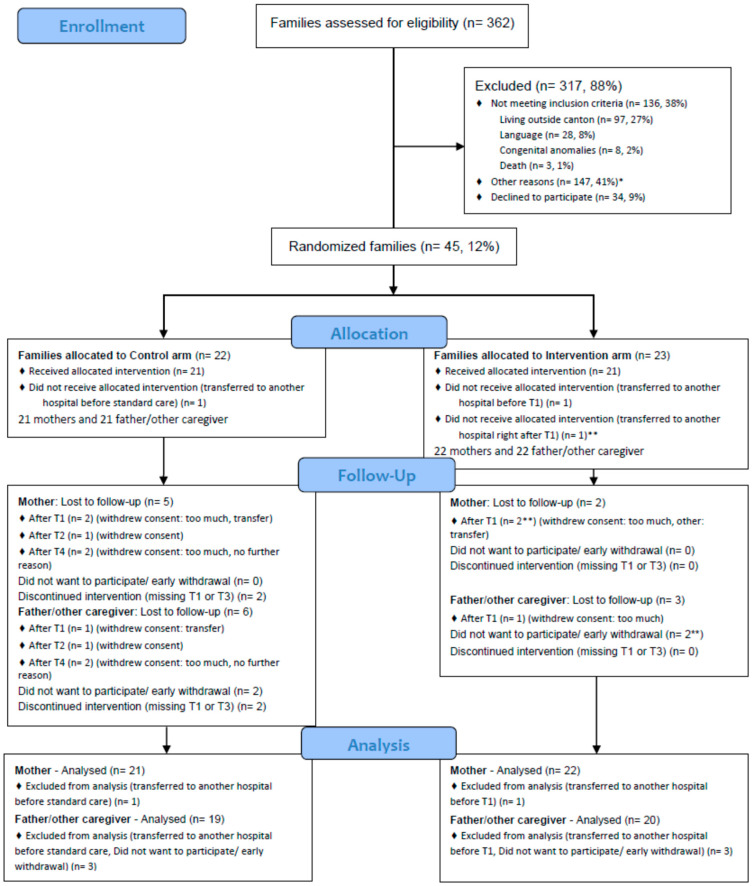
CONSORT Flow diagram [28]. * transferred to another hospital during the recruiting process (n = 97, 27%), discharge during the recruiting process (n = 3, 1%), recruiting stop (n = 20, 6%), completeness of gestational group (n = 15, 4%), parental non-decision (n = 4, 1%), mother or children very sick (n = 7, 2%), parental non-compliance (n = 1, 0.3%). ** includes family transferred to another hospital after T1.

Figure 1 shows the flow diagram of the participants throughout the study period. 

Of the 362 families assessed for eligibility, 136 (38%) did not meet the pre-defined inclusion criteria, and 147 (41%) were not eligible because of other reasons. Two-thirds of these not-eligible participants were transferred to another hospital or discharged during the recruiting process. Eventually, 79 (22%) families were eligible for the TtH model. In total, 34 (9%) did prefer not to participate and 45 (12%) were included in the study.

#### 3.1.2. Study Population and Baseline Characteristics

Participants (n = 45) and non-participants (n = 34) did not differ with respect to sociodemographic characteristics except (see Supplementary Materials Tables S1 and S2), for the geographical area where people lived and fathers’ nationality. Non-participants more often lived in rural areas (44% vs. 18%) whereas participants more often lived in urban regions (56% vs. 41%). Participating fathers more often were Swiss (76% vs. 64%) while non-participating fathers more frequently had a foreign background (27% vs. 11%).

Table 1 summarizes the sociodemographic characteristics of mothers and fathers in both groups and Table 2 summarizes baseline outcome data for depression, anxiety, post-traumatic stress disorders, parenting stress and self-efficacy for mothers and fathers in both groups.

Continuous variables are presented with a mean (M) and standard deviation (SD), or median, lower quartile (lq) and upper quartile (uq) or range [min; max]. Categorical variables are presented with the number (n) and percentage (%) of participants. If there are missing data, the number of missing observations is shown.

Sociodemographic characteristics were similar in both groups. Most of the couples were married and all were living together with their partner. One couple consisted of two women. Most mothers and fathers in both groups were Swiss and German-speaking. More than two-thirds had a yearly average family income above the average of 80,000 Swiss francs. Mothers in the IG worked at a higher (M = 65.5, SD = ± 13.7) average occupation percentage than mothers in the CG (M = 49.5, SD = ± 19.8).

At the time of birth, the included premature infants (N = 47) had a mean gestational age (GA) of 29.1 ± 3.1 weeks (median = 29; IQ-range = 26; 32), a mean birth weight of 1248.7 g ± 514.2 (median = 1205.0 g; IQ-range = 840.0; 1580.0) and a mean birth length of 39.0 cm ± 5.4 (median = 40.0 cm; IQ-range = 34.0; 43.0). 47% (n = 22) of the premature infants were female. There was no difference between groups. Preterm infants in the CG (n = 22) had a mean GA at birth of 29.2 weeks ± 3.0 (median = 30.0; IQ-range = 26.0; 32.0) versus IG (n = 25) 29.0 weeks ± 3.2 (median = 28.5; IQ-range = 26.0; 32.3), a mean birth weight of 1287.5 ± 524.5 g (median = 1287.5; IQ-range = 820.0; 1593.8) versus IG 1214.6 ± 513.3 g (median = 1200.0; IQ-range = 810.0; 1565.0) and a mean birth length of 39.1 ± 5.5 cm (median = 41.0 cm; IQ-range = 34.5; 42.1) versus IG 38.8 ± 5.5 cm (median = 40.0 cm; IQ-range = 33.5; 43.3). 36% (n = 8) of infants in the CG were female versus 56% (n = 14) in the IG. Mean overall length of hospital stay (LOS) was 66.8 days ± 46.3 (median = 60.0 days; IQ-range = 29.5; 94.5) In the CG, preterm infants had a mean LOS of 62.9 days ± 40.6 (median = 52.5 days; IQ-range = 31.3; 97.0) while the mean LOS in the IG was 70.4 (days) ± 51.8 (median = 60.0 days; IQ-range = 28.5; 94.5).

There was no difference regarding the average corrected age at discharge between CG and IG. At discharge, the average corrected age of preterm infants overall (n = 41) was 40 weeks GA M = −8 days; SD = ± 30 (median = −18 days; IQ-range = −25; −1); for the CG (n = 20), the average corrected age was 40 weeks GA M = −11 days; SD = ±25 (median = −21 days; IQ-range = −29; 5); and for the IG (n = 21), the average corrected age was 40 weeks GA M = −5 days; SD = ± 36 (median = −14 days; IQ-range = −24; −3).

The baseline outcome data of fathers did not differ between groups, while the baseline scores in mothers of the following outcomes varied: state AI, TOPSE total score and sub-scores “pressure”, “self-acceptance”, “learning and knowledge”, PSI-SF total score and sub-score “parental distress”.

#### 3.1.3. Study Burden

Study burden measured six months after hospital discharge (T5) did not differ between IG and CG. For mothers in the CG, the crude mean was 3.7 (CI = 2.7 to 4.7) and for mothers in the IG, it was 3.0 (CI = 2.0 to 3.9) with an adjusted mean difference of −0.65 (CI = −1.93 to 0.63, *p* = 0.308). For fathers in the CG, the crude mean was 4.1 (CI = 2.9 to 5.2) and for fathers in the IG, it was 3.3 (CI = 2.2 to 4.4) with an adjusted mean difference of −0.63 (CI = −2.15 to 0.89, *p* = 0.402).

### 3.2. Mental Health Outcomes

#### 3.2.1. Depression, Anxiety, and Post-Traumatic Stress Disorders

**Figure 2:**
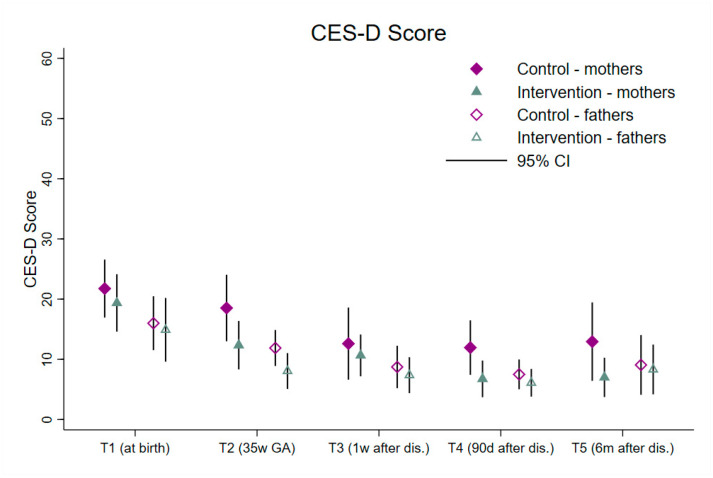
Course of depression (CES-D) scores from birth until six months after preterm infants’ discharge in mothers and fathers of the CG and the IG.

Table 3 presents the comparison between CG and IG regarding depression, anxiety and post-traumatic stress disorders for mothers and fathers. Mothers’ and fathers’ scores for depression, anxiety and PTSD progressively decreased until T4 in the CG and the IG. Mothers’ scores in the CG increased again between T4 and T5. Fathers also experienced a minimal increase in depression scores between T4 and T5 in CG and IG and for PTSD in the CG. Figure 2 illustrates the progression of depression scores in mothers and fathers of both groups. Additional graphs for anxiety and PTSD are included in the Supplementary Materials (Figures S1–S4). No differences between CG and IG were found except for depression in mothers at T2 (*p* = 0.042) and T5 (*p* = 0.027) and state anxiety in fathers at T2 (*p* = 0.016).

#### 3.2.2. Parenting Stress and Self-Efficacy

Table 4 presents the comparison between CG and IG regarding parenting stress and self-efficacy for mothers and fathers. Total parenting stress and the parental distress sub-score in mothers differed between CG and IG at baseline (*p* = 0.033, respectively, *p* = 0.32) but did not differ over time. PSI total score in mothers of the IG increased from T1 to T2 and steadily decreased until T5. Mothers in the CG showed a decrease in total parenting stress scores and sub-scores parental distress and parent–child dysfunctional interaction until T4 with, again, an increase from T4 to T5, while mothers in the IG showed a decrease in the total score and the sub-scores until T5. In the difficult child sub-score, an increase from baseline until T3 with a decrease afterward occurred in both groups.

Fathers’ parenting stress was higher at baseline with a decrease over time in both groups. Total parenting stress and the parent–child sub-score dysfunctional interaction differed at T2 with lower scores in the IG (*p* = 0.044, respectively, *p* = 0.012). 

Mothers’ self-efficacy total and sub-scores were higher in the IG, although not significantly different from the CG. In both groups, mothers’ scores increased over time from T1 until T5, except in the pressure sub-score, where an unstable pattern is shown. 

Fathers’ self-efficacy totals and sub-scores were higher in the IG, with a significant difference in the empathy and understanding sub-score at T3 (*p* = 0.025) and self-acceptance at T2 (*p* = 0.004) and an increase in total and all sub-scores over time.

### 3.3. Ancillary Analysis

Per-protocol analysis did not differ from the full analysis. Results are shown in the Supplementary Materials Tables S3–S6. Intraclass correlations for mothers and fathers between timepoints and Pearson’s correlation between fathers and mothers are shown in Supplementary Materials Tables S7 and S8. The information from the ancillary analysis is important to plan a full-scale RCT.

## 4. Discussion

This pilot randomized controlled trial aimed at verifying the feasibility of the implementation of the study design. Additionally, it generated preliminary results regarding the potential influence of the Transition to Home interventions on parental mental health and self-efficacy.

### 4.1. Feasibility of the Trial

#### 4.1.1. Recruitment

The aim of recruiting 46 families (23 per group) was almost reached with 22 families in the CG and 23 families in the IG. Nevertheless, the implementation of this pilot RCT revealed some recruitment challenges. The recruitment period was considerably longer than anticipated, i.e., it took 17 months instead of the planned six months to include the intended sample. One-third of eligible participants were transferred to another hospital during the recruitment process due to a lack of NICU beds. Parents, therefore, could not be asked to participate in the study. This high number of transfers was unexpected, although it is a common issue and recruiting barrier in this population [51]. To successfully recruit higher numbers of participants within a given period, this issue must be considered in a full-scale RCT. Participation of non-tertiary hospitals in an intervention study is one option to facilitate the recruitment of participants who are transferred to these hospitals [51].

The low refusal rate of 10.7%, in contrast, indicates a high acceptance of the study’s aims. Parents seem to see the potential benefits of the new model of care and are thus open to participation. The involvement of a large and well-trained recruitment team of nurses, who spent time explaining the study in detail and answering questions from the parents, may have supported the willingness for study participation. This proved to be an effective strategy and should be kept in the future.

In this pilot RCT, we reached fewer families living in rural areas. The comparison of participants to non-participants also showed that more fathers had a foreign background in the group of families that denied participation. Perhaps this reservation to participate can be explained by cultural and/or language barriers or the intervention not sufficiently addressing the needs of possibly socially disadvantaged individuals or those with migrant backgrounds [52]. The acceptance of the intervention in this target group and thus their participation could possibly be increased by including affected families in the further development of the intervention and the recruitment strategy [52]. 

Furthermore, we excluded families who did not write and speak German, French or English. The reasons were the limited availability of valid instruments in other languages and the lack of funds for the inclusion of an interpreter service. In their review of strategies on how to improve recruitment strategies for “hard-to-reach” populations, Bonevski et al. [52] describe common issues of restrictive eligibility criteria (e.g., language) that lead to exclusion of possible socially disadvantaged people, especially if self-administered surveys are used. 

Recommendations to overcome such issues include flexible and participant-circumstance-adapted data collection methods (e.g., face-to-face data collection), simplified data collection materials, the use of short-form measurements or investment in interpreter services for foreign-language-speaking individuals [51]. 

Finally, a group of about 100 families were not eligible because they were not living in the state of the university hospital location. This eligible criterion was applied in this pilot trial because of economic and organizational reasons. A qualitative study with healthcare professionals evaluating the implementation process of the TtH model showed that the distances covered, and the time resources invested by APNs, were already extensive even when restricted to one state [18]. To increase the potential number of participants in a full-scale trial while ensuring the feasibility of its implementation, an adaptation of the intervention delivery methods is suggested. Enhancing services through online consultations or embedding e-health methods are opportunities to support families over long distances [53,54,55]. Such methods are still novel, and few have been previously tested [54].

#### 4.1.2. Follow-Up and Study Burden

The loss-to-follow-up rate was generally low, although it was higher in the CG than in the IG. In contrast to the high rate of non-eligible participants due to transfer to a non-tertiary hospital, the drop-out rate due to a transfer was low. However, it must be considered in the calculation of the sample size for a full-scale trial. 

An individual high burden was provided as the main reason for discontinuation. The study burden, however, was low to moderate, with higher scores in the control group and among fathers. A qualitative study evaluating the TtH model from the parents’ perspectives showed that fathers felt less intensely involved and cared and they experienced multiple family burdens [19]. This is a possible explanation for why more fathers than mothers had withdrawn in our study. This explanation is supported by Davison et al., who attributed the low participation of fathers in pediatric studies to their high time constraints and lack of external motivation [56]. They recommend targeted recruitment strategies, such as personal interviews and specific written materials for fathers, to increase their participation and retention in studies. The above-suggested enhancement of the TtH model with new care delivery methods may also contribute to improved engagement of fathers.

#### 4.1.3. Outcome Measurements Used

A large set of instruments measuring similar aspects of mental health was included in the pilot study. Technically all instruments proved to be applicable at different points in time. Ideally, however, a future full-scale trial should focus on instruments that have strong psychometric properties, are suitable for the population under study and are multi-lingually available [57].

Based on the results obtained in our study, the Center for Epidemiological Studies Depression Scale (CES-D) and the State-Trait Anxiety Inventory (STAI) seem appropriate to monitor the development of depression and anxiety. Both are key aspects of mental health in parents of preterm infants and must be included [6,58]. The measured decrease in depression and anxiety between baseline and T3 and between T4 and T5 seems plausible. Both instruments are also available in a variety of languages, which makes them attractive when considering the inclusion of a wider population. Similarly, the PCL-5 checklist for post-traumatic stress and the Tool to measure Parenting Self-Efficacy covered relevant aspects that are not included in any of the other instruments used. The two questionnaires should thus be implemented in a future study. In contrast, the Parenting Stress Index Short Form (PSI–SF) was associated with some difficulties. As the results showed, participants were unable to fully comply with the questionnaire, which resulted in missing data for various sub-dimensions of the PSI-SF. At time T1, for example, only 28% of the mothers and 21% of the fathers were able to provide a complete response. To some extent, the PSI-SF seems to not be well suited for parents of preterm infants. Examples of unsuitable questions are “Sometimes my child does things that bother me just to be mean”; “Think carefully and count the number of things which your child does that bothers you. For example, dawdles, refuses to listen, overactive, cries, interrupts, fights, whines, etc.”. Furthermore, it can be argued that the PSI-SF is related to depression and anxiety. These two dimensions will surely have an influence on parenting stress, but they are measured with individual instruments. Consequently, we suggest dropping the PSI-SF for a future RCT.

### 4.2. Exploration of Effects on Parental Mental Health and Self-Efficacy

Generally, the results of our pilot study indicate that negative mental health outcomes decreased in both the IG and CG from birth until three months after the infants’ discharge from the hospital. Following this, a slight increase until six months after discharge was observed in the CG. A possible explanation for the increase in the CG could be more challenging infant behaviors or difficult parent–child interactions as the infants grow older, combined with the lack of support available to the intervention group (IG). Similar findings were reported by Treyvaud et al. [6], and Sandnes et al. [58]. Another factor may be an increased parental exhaustion resulting from the cumulative experiences of the preceding months. Self-efficacy improved over time in both groups. These initial findings are in line with our expectations, indicating a positive impact of the new model of care. The largest improvement was found during hospitalization, from birth until the age of 35 weeks GA. Possibly this can be attributed to the high-quality of standard care at our university hospital and the new interventions. Hirter et al. [49] found similar effects due to measures enhancing the mother–infant bonding. Investigating the impact of the new model after discharge must therefore be the focus of a future trial. The results of this pilot study are not yet conclusive for this period. 

Interestingly, the study revealed differences in some outcomes at baseline—although the IG and CG did not differ regarding their sociodemographic characteristics. This can suggest that the definition of the baseline needs to be reconsidered in that the baseline must be shifted to an earlier point in time to reduce the risk of confounding factors that already have an influence on mental health. Recording baseline before birth may be an option. Alternatively, more details on the sample such as medical information regarding the child or the birth must be collected and considered in the evaluation. 

Baseline scores were generally lower for fathers than for mothers and showed a minor improvement over time. The effect sizes in outcomes reported by fathers after discharge were small; effect sizes only increased to moderate or high at 35 weeks GA. It remains unclear at this point why the preliminary results did not indicate a stronger impact on fathers, whereas a clear trend was shown for mothers. However, this poses a potential statistical issue for a future trial. Small effect sizes demand a large sample size to ensure that the study is not underpowered. Given the lack of scientific studies addressing fathers, it would be interesting to specifically investigate the impact of the new model of care on fathers. At the same time, it must be questioned whether the differentiation between mothers and fathers is appropriate. Since the “Transition to Home” model emphasizes its family centration, this family aspect should be evaluated when assessing the impact of the model. Therefore, it would not be sufficient to only focus on mothers. Nonetheless, it can be assumed that in many cases, one person is the primary caregiver and thus has more interaction within the new model of care. The definition of a primary caregiver would also allow different parent constellations to be included equally. For a future trial, this aspect must be discussed and a focus on a primary caregiver should be considered along with other options to assess the impact of the model of care on other family members or the entire family unit.

### 4.3. Limitations, Lessons Learned and Recommendations for Future Research

As expected, this pilot RCT is associated with limitations that need to be dispelled or considered, respectively, in a future evaluation of our intervention. Based on the results, the required final sample size can be determined. However, for some parameters, the effect size for fathers after discharge was very small such that a large sample size for fathers is expected, which is not so for mothers. As mentioned above, the differentiation between fathers and mothers should therefore be reconsidered. 

The sample of this pilot study was biased in that it primarily included well-educated parents with a medium to high yearly family income. It is known that preterm birth can be associated with low socioeconomic status [59] and it is therefore hypothesized that an intervention like TtH can have a positive effect on socially deprived families. Therefore, a future sample should be more diverse with respect to the socioeconomic status of the participants to also address such research questions. Providing the intervention and the evaluation instruments, respectively, in more languages will allow more families, e.g., with a migration background, to be included. Potentially, the inclusion of a more diverse sample will require the actual intervention to be adjusted or amended to meet any further needs that were not yet required by the sample of the pilot RCT. To improve the recruitment process, especially in terms of accelerating it, the inclusion of more hospitals should be considered. Slightly reducing the selection of evaluation instruments as outlined above will also streamline a future study design, although it should be noted that the study burden was low for both the intervention and the comparison group. 

## 5. Conclusions

This pilot RCT was necessary to establish an appropriate framework for evaluating a complex intervention like the TtH model of care; the study served as a proof of concept for the assessment scheme, demonstrating its viability. The results indicate that the overall structure of the RCT is appropriate. Its feasibility is given with respect to the processes and instruments used. Minor adjustments in the study design are suggested, specifically to ensure the inclusion of a more diverse sample in a future RCT.

## Figures and Tables

**Table 1:** Sample characteristics in total and by group for mothers and fathers.

		Mothers			Fathers	
	All	Control Group	Intervention Group	All	Control Group	Intervention Group
	n (%) or Median (IQ-Range) or Mean ± sd	n (%) or Median (IQ-Range) or Mean ± sd	n (%) or Median (IQ-Range) or Mean ± sd	n (%) or Median (IQ-Range) or Mean ± sd	n (%) or Median (IQ-Range) or Mean ± sd	n (%) or Median (IQ-Range) or Mean ± sd
	N = 43	n = 21	n = 22	N = 39	n = 19	n = 20
Age	32.0 (31.0; 35.0)	32.0 (30.0; 34.5)	32.0 (31.0; 35.3)	35.0 (33.0; 39.0)	35.0 (33.0; 40.0)	34.5 (31.5; 38.0)
	32.7 ± 2.8	32.2 ± 2.8	33.2 ± 2.7	36.2 ± 6.6	36.1 ± 5.0	36.4 ± 7.9
Marital status						
Married	31 (72%	17 (81%)	14 (64%)	28 (72%)	15 (79%)	13 (65%)
Unmarried	12 (28%)	4 (19%)	8 (36%)	11 (28%)	4 (21%)	7 (35%)
Nationality						
Swiss	37 (86%)	20 (95%)	17 (77%)	34 (87%)	18 (95%)	16 (80%)
German	3 (7%)	1 (5%)	2 (9%)	4 (10%)	1 (5%)	3 (15%)
Italian				1 (3%)	0 (0%)	1 (5%)
Macedonian	1 (2%)	0 (0%)	1 (5%)			
Other	2 (5%)	0 (0%)	2 (9%)			
Mother tongue						
German	34 (79%)	18 (86%)	16 (73%)	34 (87%)	18 (95%)	16 (80%)
French	2 (5%)	1 (5%)	1 (5%)	1 (3%)	1 (5%)	0 (0%)
Italian	2 (5%)	1 (5%)	1 (5%)	1 (3%)	0 (0%)	1 (5%)
Macedonian	1 (2%)	0 (0%)	1 (5%)	1 (3%)	0 (0%)	1 (5%)
Portuguese	1 (2%)	0 (0%)	1 (5%)			
Other	3 (7%)	1 (5%)	2 (9%)	2 (5%)	0 (0%)	2 (10%)
Living in Switzerland						
Since birth	35 (81%)	19 (90%)	16 (73%)	34 (87%)	18 (95%)	16 (80%)
Since > 20 years	2 (5%)	1 (5%)	1 (5%)	2 (5%)	0 (0%)	2 (10%)
Since > 5 years	2 (5%)	0 (0%)	2 (9%)	1 (3%)	0 (0%)	1 (5%)
Since > 2 years	2 (5%)	0 (0%)	2 (9%)	1 (3%)	0 (0%)	1 (5%)
Since < 2 years	2 (5%)	1 (5%)	1 (5%)	1 (3%)	1 (5%)	0 (0%)
Occupation						
Full time	10 (23%)	4 (19%)	6 (27%)	26 (67%)	14 (74%)	12 (60%)
Part-time	23 (53%)	11 (52%)	12 (55%)	10 (26%)	5 (26%)	5 (25%)
Not employed	10 (23%)	6 (29%)	4 (18%)	3 (8%)	0 (0%)	3 (15%)
Occupation percentage	60.0 (40.0; 80.0) *	40.0 (39.0; 70.0) *	60.0 (60.0; 80.0) *	80.0 (65.0; 87.5)	80.0 (65.0; 87.5)	80.0 (65.0; 87.5)
(if part-time)	57.5 ± 18.5 *	49.5 ± 19.8 *	65.5 ± 13.7 *	77.2 ± 11.5	77.0 ± 12.0	77.5 ± 12.6
Educational level						
Elementary school	3 (7%)	0 (0%)	3 (14%)			
Apprenticeship	13 (30%)	10 (48%)	3 (14%)	10 (26%)	4 (21%)	6 (30%)
College of higher education	9 (21%)	3 (14%)	6 (27%)	9 (23%)	8 (42%)	1 (5%)
University degree	16 (37%)	6 (29%)	10 (46%)	18 (46%)	7 (37%)	11 (55%)
other	1 (2%)	1 (5%)	0 (0%)	2 (5%)	0 (0%)	2 (10%)
Family yearly income						
40,000–60,000 SFr. **	4 (9%)	2 (10%)	2 (9%)			
60,000–80,000 SFr. **	7 (16%)	4 (19%)	3 (14%)			
80,000–100,000 SFr. **	6 (14%)	0 (0%)	6 (27%)			
>100,000 SFr. **	24 (56%)	13 (62%)	11 (50%)			
Pregnancy disease	22 (51%)	11 (52%)	11 (50%)			
Diabetus	1 (2%)	0 (0%)	1 (5%)			
Hypertension	8 (19%)	4 (19%)	4 (18%)			
Pre-eclampsia	14 (33%)	9 (43%)	5 (23%)			
Other disease	10 (23%)	4 (19%)	6 (27%)			
Mode of delivery						
Vaginal delivery	5 (12%)	3 (14%)	2 (9%)			
Planned caesarean	10 (23%)	3 (14%)	7 (32%)			
Unplanned caesarean	28 (65%)	15 (71%)	13 (59%)			
Multiple births	4 (9%)	1 (5%)	3 (14%)			
Preterm infant is first child	23 (53%)	10 (48%)	13 (59%)			
Total no of children in family						
1	21 (49%)	9 (43%)	12 (55%)			
2	17 (40%)	10 (48%)	7 (32%)			
3	5 (12%)	2 (10%)	3 (14%)			

* *p*-value < 0.05; ** SFr. = Swiss Francs.

**Table 2:** Baseline outcome data in total and by group for mothers and fathers.

		Mothers				Fathers		
	All	Control Group	Intervention Group	Missing	All	Control Group	Intervention Group	Missing
	n (%) or Median (IQ-Range) or Mean ± sd	n (%) or Median (IQ-Range) or Mean ± sd	n (%) or Median (IQ-Range) or Mean ± sd	n (%)	n (%) or Median (IQ-Range) or Mean ± sd	n (%) or Median (IQ-Range) or Mean ± sd	n (%) or Median (IQ-Range) or Mean ± sd	n (%)
Total N	N = 43	n = 21	n = 22		N = 39	n = 19	n = 20	
CES-D	20.0 (10.5; 29.5)	20.0 (14.0; 29.0)	17.5 (9.8; 30.3)	2 (5%)	12.0 (8.0; 21.0)	14.0 (9.0; 21.0)	11.5 (6.3; 20.5)	0 (0%)
	20.7 ± 10.5	22.3 ± 10.3	19.4 ± 10.8	2 (5%)	15.3 ± 10.0	15.8 ± 8.8	14.9 ± 11.3	0 (0%)
STAI *	81.0 (66.0; 95.5)	91.0 (74.0; 105.0)	77.0 (57.0; 92.0)	2 (5%)	74.0 (64.0; 84.0)	76.0 (68.0; 83.0)	67.5 (57.0; 86.8)	0 (0%)
	82.8 ± 20.6	89.3 ± 21.9	77.1 ± 18.1	2 (5%)	75.6 ± 18.9	76.5 ± 13.5	74.8 ± 23.2	0 (0%)
State AI *	43.0 (34.5; 50.0)	47.0 (41.0; 55.0)	39.5 (32.3; 47.0)	2 (5%)	37.0 (33.0; 46.0)	37.0 (36.0; 44.0)	37.0 (31.3; 49.3)	0 (0%)
	43.4 ± 12.0	47.5 ± 13.1 **	40.0 ± 10.0 **	2 (5%)	40.2 ± 10.8	39.5 ± 7.8	40.8 ± 13.2	0 (0%)
Trait AI *	40.0 (31.0; 45.0)	41.0 (33.0; 48.0)	39.5 (28.8; 45.0)	2 (5%)	34.0 (30.0; 40.0)	37.0 (33.0; 40.0)	31.5 (26.0; 40.3)	0 (0%)
	39.3 ± 9.7	41.8 ± 9.9	37.1 ± 9.2	2 (5%)	35.5 ± 9.2	37.1 ± 7.3	34.0 ± 10.7	0 (0%)
PTSD *	14.0 (7.0; 20.5)	13.0 (10.0; 29.0)	14.5 (5.3; 20.0)	2 (5%)	11.0 (3.0; 17.0)	12.0 (7.0; 18.0)	10.0 (2.3; 15.5)	0 (0%)
	15.6 ± 12.2	19.3 ± 15.1	12.4 ± 8.1	2 (5%)	11.8 ± 9.2	12.5 ± 6.9	11.1 ± 11.1	0 (0%)
Total N	N = 47	n = 22	n = 25		N = 42	n = 20	n = 22	
TOPSE *	238.5 (218.8; 261.0)	234.0 (211.0; 248.0)	243.0 (231.0; 268.0)	5 (11%)	243.0 (220.8; 266.5)	249.0 (211.0; 269.5)	242.0 (226.0; 262.0)	2 (5%)
	237 ± 36	223 ± 41	248 ± 27	5 (11%)	242.3 ± 31.0	242.6 ± 36.0	242.0 ± 27.2	2 (5%)
emotion and	47.0 (43.0; 54.0)	45.5 (39.5; 54.0)	50.0 (46.0; 55.0)	2 (4%)	48.0 (45.8; 53.3)	49.5 (45.5; 53.8)	48.0 (45.5; 53.5)	0 (0%)
affection *	47 ± 10	46 ± 9	48 ± 10	2 (4%)	48.0 ± 8.8	47.1 ± 11.3	48.7 ± 5.8	0 (0%)
empathy and	46.0 (37.5; 53.5)	43.0 (32.5; 52.0)	50.0 (39.5; 54.5)	2 (4%)	47.0 (38.0; 54.0)	46.0 (34.0; 56.0)	47.5 (38.0; 52.8)	1 (2%)
understanding *	44 ± 11	42 ± 12	46 ± 10	2 (4%)	44.1 ± 12.3	43.3 ± 14.1	44.9 ± 10.7	1 (2%)
pressures *	46.0 (33.0; 56.0)	44.5 (29.3; 52.0) **	53.0 (35.0; 58.5) **	2 (4%)	51.5 (41.8; 55.3)	52.0 (42.5; 55.8)	51.0 (41.0; 55.8)	0 (0%)
	44 ± 13	41 ± 13	48 ± 12	2 (4%)	49.0 ± 9.4	48.8 ± 10.3	49.3 ± 8.8	0 (0%)
self-	50.0 (47.0; 55.0)	49.0 (35.3; 53.0) **	53.0 (48.5; 56.0) **	2 (4%)	53.0 (47.0; 58.0)	52.5 (47.0; 57.8)	53.0 (46.8; 58.3)	0 (0%)
acceptance *	49 ± 9	45 ± 11	52 ± 6	2 (4%)	50.8 ± 7.8	50.9 ± 8.1	50.6 ± 7.7	0 (0%)
learning and	51.0 (47.0; 53.8)	47.5 (42.0; 53.0) **	53.0 (49.0; 55.0) **	3 (6%)	49.5 (44.0; 53.3)	50.0 (43.0; 54.8)	47.0 (44.0; 53.0)	0 (0%)
knowledge *	50 ± 6	47 ± 7	52 ± 4	3 (6%)	48.9 ± 6.3	49.3 ± 6.9	48.5 ± 6.0	0 (0%)
PSI-SF *	62.0 (52.8; 77.8)	76.0 (59.5; 95.0) **	59.0 (46.5; 72.5) **	13 (28%)	66.0 (51.5; 83.5)	69.0 (53.8; 87.8)	63.0 (50.0; 80.5)	9 (21%)
	67 ± 18	75 ± 18	62 ± 17	13 (28%)	68.9 ± 19.4	72.4 ± 20.0	65.6 ± 18.8	9 (21%)
Parental	23.0 (13.0; 31.0)	28.0 (16.0; 32.0) **	19.0 (12.3; 25.5) **	4 (9%)	20.0 (13.0; 27.0)	23.0 (14.3; 27.8)	19.5 (12.8; 23.0)	0 (0%)
distress *	22 ± 8	25 ± 9	20 ± 7	4 (9%)	20.9 ± 7.7	22.4 ± 7.9	19.5 ± 7.5	0 (0%)
Parent–child	18.5 (16.0; 24.5)	21.0 (16.0; 31.5)	17.0 (16.0; 21.8)	5 (11%)	19.0 (16.0; 26.0)	19.5 (16.0; 27.8)	19.0 (16.0; 24.0)	3 (7%)
dys. int. *	21 ± 7	24 ± 8	19 ± 6	5 (11%)	21.4 ± 6.2	21.8 ± 6.7	21.1 ± 5.8	3 (7%)
Difficult	22.0 (18.0; 26.5)	21.0 (19.0; 34.0)	22.0 (18.0; 25.5)	13 (28%)	24.0 (19.5; 30.0)	27.0 (19.8; 32.3)	23.0 (19.0; 30.0)	9 (21%)
child *	24 ± 7	25 ± 8	23 ± 7	13 (28%)	26.0 ± 7.8	26.8 ± 8.3	25.4 ± 7.5	9 (21%)

* imputed scores; ** *p*-value < 0.05.

**Table 3:** Results of repeated measures mixed-effects linear regression models adjusted for stratification factors with random intercept for subjects accounting for correlation of depression, anxiety and post-traumatic stress disorders between timepoints; an interaction between randomization groups and time was included in the model.

			Mother				Father		
Outcome		Crude Mean (95%-CI) CG	Crude Mean (95%-CI) IG	Adjusted Mean Difference (95%-CI) IG-CG	Effect Size	Crude Mean (95%-CI) CG	Crude Mean (95%-CI) IG	Adjusted Mean Difference (95%-CI) IG-CG	Effect Size
	T2	18.5 (13.0 to 24.1)	12.3 (8.3 to 16.4)	−5.00 (−9.81 to −0.18) **	0.61	11.9 (8.9 to 14.9)	8.1 (5.1 to 11.0)	−3.35 (−7.07 to 0.38)	0.64
CES-D	T3	12.6 (6.6 to 18.6)	10.7 (7.2 to 14.1)	−0.57 (−5.95 to 4.80)	0.20	8.7 (5.2 to 12.3)	7.4 (4.4 to 10.4)	−0.82 (−4.65 to 3.02)	0.22
	T4	11.9 (7.4 to 16.5)	6.8 (3.7 to 9.8)	−4.21 (−8.93 to 0.50)	0.65	7.5 (5.0 to 10.0)	6.1 (3.8 to 8.4)	−0.97 (−4.75 to 2.80)	0.30
	T5	12.9 (6.4 to 19.5)	7.0 (3.7 to 10.3)	−5.77 (−10.89 to −0.64) **	0.59	9.1 (4.1 to 14.0)	8.3 (4.2 to 12.4)	−0.29 (−4.20 to 3.61)	0.09
	T2	83.8 (71.5 to 96.0)	71.2 (62.3 to 80.1)	−4.14 (−13.88 to 5.59)	0.56	75.1 (68.7 to 81.5)	65.3 (58.0 to 72.6)	−8.37 (−17.02 to 0.28)	0.71
STAI *	T3	74.3 (60.8 to 87.8)	66.0 (59.3 to 72.6)	0.18 (−10.45 to 10.82)	0.39	66.5 (59.3 to 73.7)	63.6 (56.5 to 70.8)	−1.64 (−10.49 to 7.22)	0.21
	T4	71.3 (60.4 to 82.1)	61.8 (53.9 to 69.6)	−1.23 (−12.55 to 10.08)	0.48	66.0 (58.3 to 73.7)	63.5 (54.9 to 72.1)	−1.48 (−10.23 to 7.27)	0.15
	T5	74.4 (60.4 to 88.3)	61.8 (53.5 to 70.1)	−4.12 (−16.20 to 7.96)	0.56	64.9 (53.9 to 76.0)	62.6 (52.8 to 72.4)	−1.37 (−10.34 to 7.60)	0.12
	T2	44.1 (37.4 to 50.8)	37.0 (31.5 to 42.4)	−3.42 (−9.76 to 2.92)	0.56	39.7 (36.5 to 43.0)	33.9 (30.3 to 37.6)	−5.82 (−10.55 to −1.08) **	0.82
State AI *	T3	37.1 (30.2 to 44.0)	33.3 (29.8 to 36.7)	0.77 (−5.40 to 6.94)	0.35	34.3 (30.2 to 38.3)	32.2 (29.0 to 35.3)	−2.31 (−7.16 to 2.54)	0.30
	T4	36.4 (30.5 to 42.2)	30.4 (26.5 to 34.4)	−1.87 (−8.49 to 4.74)	0.57	34.3 (30.3 to 38.4)	32.1 (27.9 to 36.2)	−2.53 (−7.32 to 2.26)	0.28
	T5	38.4 (30.6 to 46.1)	30.7 (26.5 to 34.9)	−3.32 (−10.37 to 3.74)	0.63	33.4 (27.6 to 39.3)	32.1 (26.6 to 37.5)	−1.49 (−6.41 to 3.43)	0.13
	T2	39.7 (33.5 to 45.9)	34.3 (29.9 to 38.6)	−1.10 (−5.30 to 3.11)	0.49	35.4 (32.1 to 38.7)	31.3 (27.0 to 35.6)	−2.18 (−6.68 to 2.32)	0.52
Trait AI *	T3	37.3 (30.6 to 44.0)	32.7 (28.8 to 36.6)	−1.03 (−5.86 to 3.81)	0.41	32.3 (28.5 to 36.1)	31.5 (26.9 to 36.1)	1.06 (−3.53 to 5.65)	0.09
	T4	34.9 (29.7 to 40.1)	31.3 (26.9 to 35.7)	0.20 (−4.82 to 5.22)	0.35	31.7 (27.8 to 35.5)	31.4 (26.8 to 36.1)	1.43 (−3.11 to 5.96)	0.03
	T5	36.0 (29.4 to 42.6)	31.1 (26.7 to 35.5)	−1.28 (−6.62 to 4.06)	0.44	31.5 (26.1 to 36.9)	30.5 (25.9 to 35.2)	0.49 (−4.14 to 5.12)	0.10
	T2	16.2 (9.1 to 23.2)	9.3 (6.3 to 12.4)	−2.77 (−7.43 to 1.89)	0.60	9.6 (6.3 to 13.0)	6.2 (3.0 to 9.4)	−2.48 (−5.73 to 0.77)	0.52
PTSD *	T3	14.2 (6.4 to 21.9)	8.1 (5.2 to 10.9)	−1.57 (−5.72 to 2.58)	0.53	7.0 (3.1 to 10.9)	7.6 (3.7 to 11.5)	1.27 (−2.08 to 4.61)	−0.08
	T4	10.5 (6.8 to 14.2)	6.8 (3.7 to 9.8)	0.88 (−3.47 to 5.23)	0.53	4.3 (1.1 to 7.5)	5.4 (2.6 to 8.2)	1.90 (−1.39 to 5.20)	−0.19
	T5	12.1 (4.7 to 19.4)	6.0 (3.6 to 8.4)	−2.27 (−6.78 to 2.24)	0.58	6.3 (1.4 to 11.2)	5.0 (2.2 to 7.8)	−0.68 (−4.08 to 2.72)	0.18

CG = Control Group; IG = Interventions Group; 95%-CI = 95% Confidence interval; effect size = Cohen’s d; * imputed scores or subscores; ** *p*-value < 0.05.

**Table 4:** Results of repeated measures mixed-effects linear regression models adjusted for stratification factors with random intercept for subjects accounting for correlation of parenting stress and self-efficacy between timepoints; an interaction between randomization groups and time was included in the model.

			Mother				Fathers		
Outcome		Crude Mean (95%-CI) CG	Crude Mean (95%-CI) IG	Adjusted Mean Difference (95%-CI) IG-CG	Effect Size	Crude Mean (95%-CI) CG	Crude Mean (95%-CI) IG	Adjusted Mean Difference (95%-CI) IG-CG	Effect Size
	T2	71.5 (62.9 to 80.1)	63.4 (56.3 to 70.5)	−8.69 (−20.30 to 2.92)	0.49	70.9 (58.5 to 83.2)	61.8 (54.9 to 68.7)	−9.27 (−18.30 to −0.25) **	0.51
PSI-SF *	T3	68.6 (60.7 to 76.5)	66.5 (59.6 to 73.4)	1.48 (−10.32 to 13.28)	0.13	68.9 (59.5 to 78.4)	64.6 (55.4 to 73.7)	−2.35 (−11.39 to 6.69)	0.23
	T4	61.4 (54.3 to 68.4)	62.4 (55.6 to 69.2)	−2.18 (−17.03 to 12.67)	−0.07	64.4 (56.6 to 72.2)	60.6 (54.1 to 67.2)	−0.32 (−9.22 to 8.57)	0.26
	T5	66.2 (56.5 to 75.9)	62.1 (55.8 to 68.4)	−5.71 (−23.47 to 12.06)	0.24	60.1 (52.0 to 68.3)	60.0 (53.6 to 66.5)	−1.43 (−10.60 to 7.74)	0.01
	T2	25.9 (21.4 to 30.4)	21.2 (18.0 to 24.3)	−2.60 (−6.42 to 1.23)	0.56	22.5 (18.7 to 26.3)	19.3 (16.4 to 22.2)	−1.73 (−4.98 to 1.52)	0.46
Parental	T3	22.9 (18.6 to 27.3)	22.2 (18.9 to 25.5)	0.49 (−3.71 to 4.68)	0.09	22.3 (18.4 to 26.3)	19.8 (15.9 to 23.6)	−0.85 (−4.18 to 2.48)	0.32
distress *	T4	20.8 (17.2 to 24.3)	20.3 (16.9 to 23.7)	1.30 (−2.82 to 5.42)	0.06	20.5 (16.6 to 24.3)	18.2 (15.5 to 20.8)	−1.01 (−4.30 to 2.28)	0.35
	T5	22.7 (18.2 to 27.3)	21.6 (17.9 to 25.4)	0.82 (−4.63 to 6.27)	0.12	20.3 (15.0 to 25.7)	18.5 (15.7 to 21.3)	−1.56 (−4.92 to 1.81)	0.23
Parent-Child	T2	20.8 (17.5 to 24.2)	19.0 (16.7 to 21.4)	−0.67 (−4.13 to 2.78)	0.30	21.4 (17.8 to 25.0)	18.8 (16.4 to 21.1)	−3.46 (−6.18 to −0.75) **	0.45
	T3	20.2 (17.4 to 22.9)	19.6 (17.6 to 21.6)	0.88 (−1.89 to 3.65)	0.11	20.9 (17.8 to 24.0)	19.3 (17.1 to 21.6)	−1.89 (−4.63 to 0.85)	0.29
dys. int. *	T4	18.3 (16.4 to 20.2)	18.4 (17.0 to 19.7)	0.57 (−2.29 to 3.44)	−0.01	19.4 (17.1 to 21.6)	18.0 (16.1 to 19.8)	−1.63 (−4.35 to 1.09)	0.33
	T5	19.3 (16.5 to 22.1)	17.8 (16.4 to 19.1)	−1.09 (−5.10 to 2.92)	0.34	17.5 (15.8 to 19.2)	17.8 (16.2 to 19.4)	−1.26 (−4.04 to 1.52)	−0.08
	T2	25.6 (22.3 to 28.9)	23.1 (20.3 to 26.0)	−2.20 (−6.94 to 2.54)	0.38	26.5 (21.7 to 31.3)	23.9 (21.1 to 26.8)	−2.88 (−6.94 to 1.17)	0.34
Difficult	T3	25.5 (22.3 to 28.6)	24.7 (22.0 to 27.4)	0.23 (−4.05 to 4.51)	0.12	25.8 (22.6 to 28.9)	25.5 (21.8 to 29.1)	−0.30 (−4.40 to 3.80)	0.04
child *	T4	23.0 (20.0 to 26.0)	23.8 (20.9 to 26.6)	−0.21 (−6.14 to 5.72)	−0.12	23.9 (20.9 to 27.0)	24.5 (21.6 to 27.3)	0.83 (−3.22 to 4.87)	−0.09
	T5	24.2 (20.4 to 28.0)	22.7 (20.1 to 25.2)	−3.57 (−10.27 to 3.13)	0.22	22.3 (19.8 to 24.7)	23.7 (20.8 to 26.6)	0.81 (−3.36 to 4.97)	−0.26
	T2	242.3 (226.9 to 257.6)	257.9 (246.3 to 269.5)	8.17 (−5.16 to 21.51)	−0.53	250.0 (234.3 to 265.7)	261.5 (251.8 to 271.2)	12.52 (−0.15 to 25.19)	−0.44
TOPSE *	T3	253.3 (236.6 to 270.1)	259.7 (247.8 to 271.6)	−0.69 (−15.93 to 14.54)	−0.21	256.4 (239.4 to 273.4)	263.7 (253.3 to 274.0)	8.95 (−3.89 to 21.79)	−0.28
	T4	256.6 (242.4 to 270.8)	259.1 (247.7 to 270.5)	−4.95 (−19.32 to 9.42)	−0.09	257.7 (245.5 to 269.9)	268.8 (259.2 to 278.3)	15.17 (2.64 to 27.71) **	−0.50
	T5	255.3 (239.1 to 271.5)	262.7 (251.5 to 273.9)	−0.20 (−16.43 to 16.04)	−0.26	262.3 (244.8 to 279.7)	269.6 (261.7 to 277.4)	15.55 (2.68 to 28.41) **	−0.30
	T2	51.1 (47.9 to 54.4)	54.7 (52.8 to 56.6)	4.27 (0.89 to 7.66) **	−0.61	50.5 (46.7 to 54.3)	52.6 (50.8 to 54.3)	1.86 (−1.21 to 4.94)	−0.35
Emotion &	T3	53.4 (50.7 to 56.1)	54.4 (52.2 to 56.5)	1.57 (−1.56 to 4.71)	−0.19	51.2 (47.2 to 55.2)	54.0 (52.2 to 55.7)	2.32 (−0.84 to 5.48)	−0.48
affection *	T4	54.4 (52.0 to 56.7)	54.5 (52.1 to 56.8)	0.39 (−2.78 to 3.56)	−0.02	53.9 (51.9 to 55.9)	54.6 (52.2 to 57.0)	0.54 (−2.58 to 3.65)	−0.16
	T5	55.3 (53.4 to 57.2)	55.8 (54.2 to 57.5)	0.68 (−1.42 to 2.78)	−0.14	53.7 (50.6 to 56.7)	54.7 (53.1 to 56.4)	1.18 (−2.03 to 4.39)	−0.23
	T2	48.9 (44.8 to 53.0)	50.5 (47.7 to 53.2)	1.32 (−2.53 to 5.17)	−0.21	46.7 (40.8 to 52.7)	49.9 (46.7 to 53.0)	3.95 (−0.78 to 8.69)	−0.33
Empathy &	T3	49.9 (45.8 to 54.0)	50.7 (47.6 to 53.9)	0.90 (−2.97 to 4.77)	−0.11	46.7 (42.0 to 51.4)	51.8 (49.1 to 54.5)	5.56 (0.70 to 10.43) **	−0.70
understanding *	T4	52.1 (49.0 to 55.2)	51.8 (49.0 to 54.6)	−0.50 (−4.14 to 3.15)	0.04	48.4 (44.2 to 52.6)	51.2 (48.1 to 54.3)	3.24 (−1.56 to 8.03)	−0.37
	T5	51.2 (47.0 to 55.3)	51.8 (49.3 to 54.4)	0.53 (−3.93 to 4.98)	−0.09	51.4 (47.6 to 55.2)	52.5 (50.6 to 54.5)	3.03 (−1.92 to 7.97)	−0.20
	T2	42.0 (36.6 to 47.5)	46.5 (41.0 to 52.0)	1.96 (−3.72 to 7.64)	−0.37	49.3 (44.6 to 54.0)	51.4 (47.3 to 55.5)	2.67 (−1.75 to 7.09)	−0.22
Pressures *	T3	46.3 (40.8 to 51.8)	48.1 (42.5 to 53.7)	−0.66 (−6.95 to 5.62)	−0.15	50.0 (43.2 to 56.8)	51.4 (47.6 to 55.1)	1.94 (−2.60 to 6.48)	−0.13
	T4	44.7 (38.9 to 50.5)	45.5 (40.1 to 51.0)	−1.39 (−7.27 to 4.50)	−0.07	52.4 (47.6 to 57.1)	54.8 (51.9 to 57.6)	2.94 (−1.54 to 7.42)	−0.31
	T5	42.9 (35.9 to 49.9)	46.9 (41.2 to 52.6)	1.48 (−5.50 to 8.45)	−0.30	51.0 (43.9 to 58.1)	53.5 (50.6 to 56.5)	3.49 (−1.13 to 8.10)	−0.26
	T2	46.5 (42.1 to 50.9)	52.5 (49.9 to 55.0)	3.61 (0.14 to 7.09) **	−0.77	50.6 (46.2 to 55.0)	55.0 (52.6 to 57.5)	5.10 (1.62 to 8.57) **	−0.62
Self-acceptance *	T3	50.2 (45.3 to 55.1)	52.9 (50.1 to 55.7)	0.90 (−2.57 to 4.38)	−0.32	51.6 (46.8 to 56.4)	54.1 (51.6 to 56.7)	2.80 (−0.75 to 6.36)	−0.35
	T4	51.4 (47.1 to 55.7)	53.6 (50.7 to 56.5)	0.08 (−2.98 to 3.14)	−0.28	52.4 (49.2 to 55.6)	55.2 (53.2 to 57.2)	3.37 (−0.14 to 6.89)	−0.52
	T5	51.8 (47.6 to 56.1)	54.2 (52.0 to 56.4)	0.12 (−2.20 to 2.43)	−0.35	53.2 (48.4 to 58.0)	55.5 (53.5 to 57.4)	3.07 (−0.53 to 6.68)	−0.35
	T2	53.6 (50.9 to 56.4)	53.8 (52.0 to 55.6)	−0.47 (−3.15 to 2.22)	−0.03	51.1 (47.9 to 54.2)	52.6 (50.2 to 55.1)	2.31 (−1.50 to 6.13)	−0.27
Learning &	T3	53.6 (50.9 to 56.3)	53.6 (51.9 to 55.4)	−0.74 (−3.37 to 1.89)	−0.01	50.3 (46.0 to 54.5)	52.4 (50.0 to 54.8)	2.73 (−1.19 to 6.64)	−0.32
knowledge *	T4	54.0 (51.3 to 56.8)	53.6 (51.7 to 55.6)	−1.01 (−3.77 to 1.76)	0.08	50.6 (46.9 to 54.4)	53.0 (49.2 to 56.8)	3.14 (−0.72 to 7.00)	−0.30
	T5	54.1 (51.8 to 56.4)	54.0 (51.7 to 56.2)	−0.91 (−3.50 to 1.68)	0.03	53.0 (50.2 to 55.8)	53.3 (50.8 to 55.8)	1.26 (−2.72 to 5.23)	−0.06

CG = Control Group; IG = Interventions Group; 95%-CI = 95% Confidence interval; effect size = Cohen’s d; * imputed scores or subscores; ** *p*-value < 0.05.

## Data Availability

All relevant data are presented in this manuscript. All the data are available from the corresponding author upon request.

## Supplementary Materials

The following supporting information can be downloaded at: https://www.mdpi.com/article/10.3390/children11101260/s1, Table S1: Comparison sociodemographic characteristics of participants with non-participants; mothers; Table S2: Comparison sociodemographic characteristics of participants with non-participants; fathers; Table S3: Outcomes per protocol set for depression, anxiety, and post-traumatic stress disorders for mothers; Table S4: Outcomes per protocol set for depression, anxiety, and post-traumatic stress disorders for fathers; Table S5: Outcomes per protocol set for parenting stress and self-efficacy for mothers; Table S6: Outcomes per protocol set for parenting stress and self-efficacy for fathers; Table S7: Intraclass correlations (ICC) for mothers and fathers for all scores between all timepoints; Table S8: Pearson between fathers and mothers for all scores all timepoints combined; Figure S1: Course of anxiety (STAI) scores from birth until six months after preterm infants’ discharge in mothers and fathers of the CG and the IG; Figure S2: Course of state anxiety scores from birth until six months after preterm infants’ discharge in mothers and fathers of the CG and the IG; Figure S3: Course of trait anxiety scores from birth until six months after preterm infants’ discharge in mothers and fathers of the CG and the IG; Figure S4: Course of post-traumatic stress disorders (PTSD) scores from birth until six months after preterm infants’ discharge in mothers and fathers of the CG and the IG.

## References

[1] Chen F., Bajwa N.M., Rimensberger P.C., Posfay-Barbe K.M., Pfister R.E. (2016). Thirteen-Year Mortality and Morbidity in Preterm Infants in Switzerland. Arch. Dis. Child.-Fetal Neonatal Ed..

[2] Sarda S.P., Sarri G., Siffel C. (2021). Global Prevalence of Long-Term Neurodevelopmental Impairment Following Extremely Preterm Birth: A Systematic Literature Review. J. Int. Med. Res..

[3] Huhtala M., Korja R., Lehtonen L., Haataja L., Lapinleimu H., Rautava P. (2014). Associations between Parental Psychological Well-Being and Socio-Emotional Development in 5-Year-Old Preterm Children. Early Hum. Dev..

[4] Schuetz Haemmerli N., Lemola S., Holditch-Davis D., Cignacco E. (2020). Comparative Evaluation of Parental Stress Experiences Up to 2 to 3 Years After Preterm and Term Birth. Adv. Neonatal Care.

[5] Kantrowitz-Gordon I., Altman M.R., Vandermause R. (2016). Prolonged Distress of Parents After Early Preterm Birth. J. Obstet. Gynecol. Neonatal Nurs..

[6] Treyvaud K. (2014). Parent and Family Outcomes Following Very Preterm or Very Low Birth Weight Birth: A Review. Semin. Fetal. Neonatal Med..

[7] Cheong J.L.Y., Burnett A.C., Treyvaud K., Spittle A.J. (2020). Early Environment and Long-Term Outcomes of Preterm Infants. J. Neural Transm..

[8] Pladys P., Zaoui C., Girard L., Mons F., Reynaud A., Casper C., The Group for Reflections on and Evaluation of the Neonatal Environment of the French Neonatal Society (2020). French Neonatal Society Position Paper Stresses the Importance of an Early Family-centred Approach to Discharging Preterm Infants from Hospital. Acta Paediatr..

[9] Ding X., Zhu L., Zhang R., Wang L., Wang T.-T., Latour J.M. (2019). Effects of Family-Centred Care Interventions on Preterm Infants and Parents in Neonatal Intensive Care Units: A Systematic Review and Meta-Analysis of Randomised Controlled Trials. Aust. Crit. Care.

[10] O’Brien K., Robson K., Bracht M., Cruz M., Lui K., Alvaro R., Da Silva O., Monterrosa L., Narvey M., Ng E. (2018). Effectiveness of Family Integrated Care in Neonatal Intensive Care Units on Infant and Parent Outcomes: A Multicentre, Multinational, Cluster-Randomised Controlled Trial. Lancet Child Adolesc. Health.

[11] Franck L.S., Waddington C., O’Brien K. (2020). Family Integrated Care for Preterm Infants. Crit. Care Nurs. Clin. N. Am..

[12] Cheng C., Franck L.S., Ye X.Y., Hutchinson S.A., Lee S.K., O’Brien K. (2021). Evaluating the Effect of Family Integrated Care on Maternal Stress and Anxiety in Neonatal Intensive Care Units. J. Reprod. Infant Psychol..

[13] Zhang Y., Jiang M., Wang S., Xiang X., He W., Du J., Hei M. (2024). Effect of Family Integrated Care on Stress in Mothers of Preterm Infants: A Multicenter Cluster Randomized Controlled Trial. J. Affect. Disord..

[14] Van Hus J., Jeukens-Visser M., Koldewijn K., Holman R., Kok J., Nollet F., Van Wassenaer-Leemhuis A. (2016). Early Intervention Leads to Long-term Developmental Improvements in Very Preterm Infants, Especially Infants with Bronchopulmonary Dysplasia. Acta Paediatr..

[15] Baraldi E., Allodi M.W., Löwing K., Smedler A.-C., Westrup B., Ådén U. (2020). Stockholm Preterm Interaction-Based Intervention (SPIBI)-Study Protocol for an RCT of a 12-Month Parallel-Group Post-Discharge Program for Extremely Preterm Infants and Their Parents. BMC Pediatr..

[16] Jeukens-Visser M., Koldewijn K., Van Wassenaer-Leemhuis A.G., Flierman M., Nollet F., Wolf M. (2021). Development and Nationwide Implementation of a Postdischarge Responsive Parenting Intervention Program for Very Preterm Born Children: The TOP Program. Infant Ment. Health J..

[17] Newnham C.A., Milgrom J., Skouteris H. (2009). Effectiveness of a Modified Mother–Infant Transaction Program on Outcomes for Preterm Infants from 3 to 24 Months of Age. Infant Behav. Dev..

[18] Schuetz Haemmerli N., Von Gunten G., Khan J., Stoffel L., Humpl T., Cignacco E. (2021). Interprofessional Collaboration in a New Model of Transitional Care for Families with Preterm Infants—The Health Care Professional’s Perspective. J. Multidiscip. Healthc..

[19] Schuetz Haemmerli N., Stoffel L., Schmitt K.-U., Khan J., Humpl T., Nelle M., Cignacco E. (2022). Enhancing Parents’ Well-Being after Preterm Birth—A Qualitative Evaluation of the “Transition to Home” Model of Care. Int. J. Environ. Res. Public Health.

[20] Als H., Butler S., Kosta S., McAnulty G. (2005). The Assessment of Preterm Infants’ Behavior (APIB): Furthering the Understanding and Measurement of Neurodevelopmental Competence in Preterm and Full-term Infants. Ment. Retard. Dev. Disabil. Res. Rev..

[21] Halbmeijer N.M., Jeukens-Visser M., Onland W., Flierman M., Van Kaam A.H., Leemhuis A. (2023). Neurodevelopmental Outcomes at Two Years’ Corrected Age of Very Preterm Infants after Implementation of a Post-Discharge Responsive Parenting Intervention Program (TOP Program). J. Pediatr..

[22] Meijssen D.E., Wolf M.J., Koldewijn K., Van Wassenaer A.G., Kok J.H., Van Baar A.L. (2011). Parenting Stress in Mothers after Very Preterm Birth and the Effect of the Infant Behavioural Assessment and Intervention Program: Parenting Stress after Preterm Birth and Intervention. Child Care Health Dev..

[23] Ulvund S.E. (2022). Early Intervention in Families with Preterm Infants: A Review of Findings from a Randomized Controlled Trial Following Children Up to 9 Years of Age. Children.

[24] Hynan M.T., Mounts K.O., Vanderbilt D.L. (2013). Screening Parents of High-Risk Infants for Emotional Distress: Rationale and Recommendations. J. Perinatol..

[25] Purdy I.B., Craig J.W., Zeanah P. (2015). NICU Discharge Planning and beyond: Recommendations for Parent Psychosocial Support. J. Perinatol..

[26] Treyvaud K., Spittle A., Anderson P.J., O’Brien K. (2019). A Multilayered Approach Is Needed in the NICU to Support Parents after the Preterm Birth of Their Infant. Early Hum. Dev..

[27] Skivington K., Matthews L., Simpson S.A., Craig P., Baird J., Blazeby J.M., Boyd K.A., Craig N., French D.P., McIntosh E. (2021). Framework for the Development and Evaluation of Complex Interventions: Gap Analysis, Workshop and Consultation-Informed Update. Health Technol. Assess..

[28] Schulz K.F., Altman D.G., Moher D., for the CONSORT Group (2010). CONSORT 2010 Statement: Updated Guidelines for Reporting Parallel Group Randomised Trials. BMJ.

[29] Eldridge S.M., Chan C.L., Campbell M.J., Bond C.M., Hopewell S., Thabane L., Lancaster G.A. (2016). CONSORT 2010 Statement: Extension to Randomised Pilot and Feasibility Trials. BMJ.

[30] Sivanandan S., Sankar M.J. (2023). Kangaroo Mother Care for Preterm or Low Birth Weight Infants: A Systematic Review and Meta-Analysis. BMJ Glob. Health.

[31] Bienstein C., Fröhlich A. (2021). Basale Stimulation in Der Pflege^®^.

[32] Maietta L., Hatch F. (2023). Kinaesthetics Infant Handling Kindliche Bewegungsfähigkeiten Entdecken, Entwickeln und Fördern.

[33] Wessel S., Manthey M., Mischo-Kelling M. (2023). Primary Nursing—Primäre Pflege: Ein Personenbezogenes Pflegesystem.

[34] Melnyk B.M., Alpert-Gillis L., Feinstein N.F., Fairbanks E., Schultz-Czarniak J., Hust D., Sherman L., LeMoine C., Moldenhauer Z., Small L. (2001). Improving Cognitive Development of Low-birth-weight Premature Infants with the COPE Program: A Pilot Study of the Benefit of Early NICU Intervention with Mothers. Res. Nurs. Health.

[35] Porz F., Podeswik A., Brinkmann V. (2010). Case Management in der Kinder- und Jugendmedizin. Qualitätsmanagement beim Aufbau und in der Begleitung von Nachsorgeeinrichtungen für schwer und chronisch kranke Kinder und Jugendliche am Beispiel des Bunten Kreises. Case Management Organisationsentwicklung und Change Management im Gesundheits- und Sozialunternehmen.

[36] Naylor M.D., Brooten D., Campbell R., Jacobsen B.S., Mezey M.D., Pauly M.V., Schwartz J.S. (1999). Comprehensive Discharge Planning and Home Follow-up of Hospitalized Elders: A Randomized Clinical Trial. JAMA.

[37] Brooten D., Kumar S., Brown L.P., Butts P., Finkler S.A., Bakewell-Sachs S., Gibbons A., Delivoria-Papadopoulos M. (1986). A Randomized Clinical Trial of Early Hospital Discharge and Home Follow-up of Very-Low-Birth-Weight Infants. N. Engl. J. Med..

[38] Tracy M.F., O’Grady E.T. (2019). Hamric and Hanson’s Advanced Practice Nursing: An Integrative Approach.

[39] (2019). Canadian Nurses Association Advanced Practice Nursing: A Pan-Canadian Framework.

[40] Bryant-Lukosius D., Spichiger E., Martin J., Stoll H., Kellerhals S.D., Fliedner M., Grossmann F., Henry M., Herrmann L., Koller A. (2016). Framework for Evaluating the Impact of Advanced Practice Nursing Roles. J. Nurs. Scholarsh..

[41] Gysin S., Sottas B., Odermatt M., Essig S. (2019). Advanced Practice Nurses’ and General Practitioners’ First Experiences with Introducing the Advanced Practice Nurse Role to Swiss Primary Care: A Qualitative Study. BMC Fam. Pract..

[42] Nugent J.K., Keefer C.H., Minear S., Johnson Lise C., Blanchard Y., Paul H. (2007). Understanding Newborn Behavior & Early Relationships: The Newborn Behavioral Observations (NBO) System Handbook.

[43] Hulley S.B., Cummings S.R., Browner W.S., Grady D.G., Newman T.B. (2013). Designing Clinical Research.

[44] Radloff L.S. (1977). The CES-D Scale: A Self-Report Depression Scale for Research in the General Population. Appl. Psychol. Meas..

[45] Spielberger C.D., Gorsuch R., Lushene R.E., Vagg P.R., Jacobs G.A. (1983). Manual for the State-Trait Anxiety Inventory.

[46] Weathers F.W., Litz B.T., Keane T.M., Palmieri P.A., Marx B.P., Schnurr P.P. The PTSD Checklist for DSM-5 (PCL-5) 2013. https://www.ptsd.va.gov/professional/assessment/documents/PCL5_Standard_form.pdf.

[47] Abidin R. (1995). Parenting Stress Index.

[48] Kendall S., Bloomfield L. (2005). Developing and Validating a Tool to Measure Parenting Self-efficacy. J. Adv. Nurs..

[49] Hirter K., Dinten-Schmid B., Avian A., Feinstein N., Spichiger E., Nelle M., Stoffel Zurcher L. (2021). Effect of the COPE Program on Self-Efficacy in Mothers of Preterm Infants: A Pretest-Posttest Quasi-Experimental Study. J. Perinat. Neonatal Nurs..

[50] StataCorp (2019). Stata Statistical Software: Release 16.

[51] Mörelius E., Olsson E., Sahlén Helmer C., Thernström Blomqvist Y., Angelhoff C. (2020). External Barriers for Including Parents of Preterm Infants in a Randomised Clinical Trial in the Neonatal Intensive Care Unit in Sweden: A Descriptive Study. BMJ Open.

[52] Bonevski B., Randell M., Paul C., Chapman K., Twyman L., Bryant J., Brozek I., Hughes C. (2014). Reaching the Hard-to-Reach: A Systematic Review of Strategies for Improving Health and Medical Research with Socially Disadvantaged Groups. BMC Med. Res. Methodol..

[53] Hynan M.T., Steinberg Z., Baker L., Cicco R., Geller P.A., Lassen S., Milford C., Mounts K.O., Patterson C., Saxton S. (2015). Recommendations for Mental Health Professionals in the NICU. J. Perinatol..

[54] Franck L.S., Gay C.L., Hoffmann T.J., Kriz R.M., Bisgaard R., Cormier D.M., Joe P., Lothe B., Sun Y. (2022). Neonatal Outcomes from a Quasi-Experimental Clinical Trial of Family Integrated Care versus Family-Centered Care for Preterm Infants in U.S. NICUs. BMC Pediatr..

[55] Lindkvist R.-M., Sjöström-Strand A., Landgren K., Johnsson B.A., Stenström P., Hallström I.K. (2021). “In a Way We Took the Hospital Home”—A Descriptive Mixed-Methods Study of Parents’ Usage and Experiences of eHealth for Self-Management after Hospital Discharge Due to Pediatric Surgery or Preterm Birth. Int. J. Environ. Res. Public Health.

[56] Davison K.K., Charles J.N., Khandpur N., Nelson T.J. (2017). Fathers’ Perceived Reasons for Their Underrepresentation in Child Health Research and Strategies to Increase Their Involvement. Matern. Child Health J..

[57] Coster W.J. (2013). Making the Best Match: Selecting Outcome Measures for Clinical Trials and Outcome Studies. Am. J. Occup. Ther..

[58] Sandnes R., Le Floch M., Riquin E., Nocus I., Müller J.B., Bacro F. (2024). Parental Stress and Mental Health Outcomes Following Very Preterm Birth: A Systematic Review of Recent Findings. J. Affect. Disord..

[59] Klumper J., Ravelli A.C.J., Roos C., Abu-Hanna A., Oudijk M.A. (2022). Deprived Neighborhoods and Spontaneous Preterm Birth: A National Cohort Study. Eur. J. Obstet. Gynecol. Reprod. Biol..

