# Supplementary material for: Evaluation of a Model of Transitional Care After Preterm Birth on Parents’ Mental Health and Self-Efficacy: A Randomized Controlled Pilot Trial
Source: Children (Basel). 2024 Oct 18;11(10):1260. doi: 10.3390/children11101260 (PMC11505660; doi:10.3390/children11101260)
Supplement: Supplementary file 1 [file children-11-01260-s001.zip › children-3246133-supplementary.pdf]

Evaluation of a Model of Transitional Care after Preterm Birth on Parents' Mental Health and Self-efficacy: A Randomized Controlled Pilot Trial

Supplementary materials

Table S1. Comparison sociodemographic characteristics of participants with non-participants; mothers

	Participant	Non-participant	p-value	Missing
	n (%) or median (IQ-range) or mean ± sd			*n (%)*
Total N	N = 45	N = 34		N = 79
Age	32.0 (31.0; 35.0)	30.5 (27.8; 35.3)	0.112	2 (3%)
Marital status			0.273	2 (3%)
married	31 (69%)	23 (68%)		
separate	0 (0%)	2 (6%)		
unmarried	12 (27%)	9 (26%)		
Living in Switzerland since			0.511	5 (6%)
since birth	35 (78%)	23 (68%)		
since >20 years	2 (4%)	2 (6%)		
since >5 years	2 (4%)	2 (6%)		
since >2 years	2 (4%)	4 (12%)		
since <2 years	2 (4%)	0 (0%)		
Nationality			0.215	3 (4%)
Swiss	37 (82%)	23 (68%)		
German	3 (7%)	1 (3%)		
Turkish	0 (0%)	1 (3%)		
Macedonian	1 (2%)	1 (3%)		
Kosovar	0 (0%)	1 (3%)		
Other	2 (4%)	6 (18%)		
Mother tongue			0.641	2 (3%)
German	34 (76%)	27 (79%)		
English	0 (0%)	1 (3%)		
French	2 (4%)	1 (3%)		
Italian	2 (4%)	0 (0%)		
Macedonian	1 (2%)	1 (3%)		
Albanian	0 (0%)	1 (3%)		
Portuguese	1 (2%)	0 (0%)		
Other	3 (7%)	3 (9%)		
Living situation			0.049	2 (3%)
alone with kid/s	0 (0%)	4 (12%)		
with Partner & Kid/s	42 (93%)	30 (88%)		
other	1 (2%)	0 (0%)		
Geographical classification			0.035	0 (0%)
urban	25 (56%)	14 (41%)		
intermediate	12 (27%)	5 (15%)		
rural	8 (18%)	15 (44%)		
Gestational age at birth (week)	29.3 (26.5; 32.8)	32.1 (29.3; 33.0)	0.059	1 (1%)
Nb birth			0.116	0 (0%)
single birth	41 (91%)	26 (76%)		
twins	4 (9%)	6 (18%)		
triplets	0 (0%)	2 (6%)		

Table S2. Comparison sociodemographic characteristics of participants with non-participants; fathers

	Participant	Non-participant	p-value	Missing
	n (%) or median (IQ-range) or mean ± sd			*n (%)*
Total N	N = 45	N = 33		N = 78
Marital status			0.537	7 (9%)
married	28 (62%)	22 (67%)		
separate	0 (0%)	1 (3%)		
unmarried	11 (24%)	9 (27%)		
Living in Switzerland since			0.777	11 (14%)
since birth	34 (76%)	22 (67%)		
since >20 years	2 (4%)	1 (3%)		
since >10 years	0 (0%)	1 (3%)		
since >5 years	1 (2%)	2 (6%)		
since >2 years	1 (2%)	1 (3%)		
since <2 years	1 (2%)	1 (3%)		
Nationality			0.013	9 (12%)
Swiss	34 (76%)	21 (64%)		
German	4 (9%)	0 (0%)		
Italian	1 (2%)	0 (0%)		
Turkish	0 (0%)	1 (3%)		
Kosovar	0 (0%)	1 (3%)		
Portuguese	0 (0%)	1 (3%)		
Other	0 (0%)	6 (18%)		
Mother tongue			0.416	7 (9%)
German	34 (76%)	23 (70%)		
English	0 (0%)	1 (3%)		
French	1 (2%)	1 (3%)		
Italian	1 (2%)	0 (0%)		
Turkish	0 (0%)	1 (3%)		
Macedonian	1 (2%)	0 (0%)		
Albanian	0 (0%)	1 (3%)		
Portuguese	0 (0%)	1 (3%)		
Other	2 (4%)	4 (12%)		
Living situation			0.228	6 (8%)
with Partner & Kid/s	38 (84%)	30 (91%)		
other	1 (2%)	3 (9%)		
Geographical classification			0.077	1 (1%)
urban	25 (56%)	14 (42%)		
intermediate	12 (27%)	5 (15%)		
rural	8 (18%)	13 (39%)		

Table S3. Outcomes per protocol set for depression, anxiety, and posttraumatic stress disorders for mothers

Outcome		Mother *Crude mean control*	*Crude mean intervention*	*Adjusted mean difference (95%-CI)* Intervention - Control	*p-value*
CES-D					
	T2 (35w GA)	18.4 (11.7 to 25.0)	12.3 (8.3 to 16.4)	-4.54 (-9.32 to 0.24)	0.063
	T3 (1w a. d.)	12.9 (5.0 to 20.8)	10.7 (7.2 to 14.1)	-0.74 (-6.76 to 5.29)	0.810
	T4 (90d a. d.)	13.5 (7.4 to 19.6)	6.8 (3.7 to 9.8)	-5.28 (-10.49 to -0.07)	0.047
	T5 (6m a. d.)	14.6 (6.8 to 22.3)	7.0 (3.7 to 10.3)	-6.10 (-11.61 to -0.59)	0.030
STAI*					
	T2 (35w GA)	85.1 (68.6 to 101.6)	71.2 (62.3 to 80.1)	-3.81 (-14.65 to 7.03)	0.491
	T3 (1w a. d.)	75.1 (57.3 to 92.8)	66.0 (59.3 to 72.6)	1.01 (-11.09 to 13.11)	0.870
	T4 (90d a. d.)	73.8 (59.5 to 88.1)	61.8 (53.9 to 69.6)	-1.90 (-14.20 to 10.40)	0.762
	T5 (6m a. d.)	76.4 (59.5 to 93.4)	61.8 (53.5 to 70.1)	-4.49 (-17.66 to 8.67)	0.503
State AI*					
	T2 (35w GA)	46.0 (37.2 to 54.8)	37.0 (31.5 to 42.4)	-3.92 (-10.96 to 3.13)	0.276
	T3 (1w a. d.)	37.5 (28.4 to 46.6)	33.3 (29.8 to 36.7)	0.88 (-6.18 to 7.95)	0.807
	T4 (90d a. d.)	38.2 (30.7 to 45.7)	30.4 (26.5 to 34.4)	-2.63 (-9.96 to 4.70)	0.481
	T5 (6m a. d.)	39.6 (30.2 to 49.0)	30.7 (26.5 to 34.9)	-3.81 (-11.56 to 3.94)	0.335
Trait AI*					
	T2 (35w GA)	39.1 (30.9 to 47.4)	34.3 (29.9 to 38.6)	-0.49 (-5.12 to 4.14)	0.835
	T3 (1w a. d.)	37.6 (28.8 to 46.3)	32.7 (28.8 to 36.6)	-0.47 (-5.89 to 4.94)	0.864
	T4 (90d a. d.)	35.6 (28.6 to 42.5)	31.3 (26.9 to 35.7)	0.13 (-5.16 to 5.42)	0.962
	T5 (6m a. d.)	36.8 (28.7 to 44.8)	31.1 (26.7 to 35.5)	-1.29 (-6.99 to 4.42)	0.659
PTSD*					
	T2 (35w GA)	17.4 (7.8 to 27.0)	9.3 (6.3 to 12.4)	-3.63 (-8.72 to 1.47)	0.163
	T3 (1w a. d.)	13.7 (3.7 to 23.7)	8.1 (5.2 to 10.9)	-1.28 (-5.68 to 3.11)	0.567
	T4 (90d a. d.)	10.3 (5.3 to 15.3)	6.8 (3.7 to 9.8)	0.84 (-3.91 to 5.60)	0.728
	T5 (6m a. d.)	12.1 (3.3 to 21.0)	6.0 (3.6 to 8.4)	-1.76 (-6.36 to 2.83)	0.452

Table S4. Outcomes per protocol set for depression, anxiety, and posttraumatic stress disorders for fathers

Outcome		Father<br>*Crude mean control*	*Crude mean intervention*	*Adjusted mean difference (95%-CI)*<br>Intervention - Control	*p-value*
CES-D					
	T2 (35w GA)	12.2 (8.4 to 16.1)	8.1 (5.1 to 11.0)	-3.76 (-7.86 to 0.34)	0.072
	T3 (1w a. d.)	9.4 (5.4 to 13.4)	7.4 (4.4 to 10.4)	-1.60 (-5.70 to 2.50)	0.444
	T4 (90d a. d.)	7.1 (4.1 to 10.0)	6.1 (3.8 to 8.4)	-0.56 (-4.66 to 3.54)	0.790
	T5 (6m a. d.)	9.5 (4.2 to 14.8)	8.3 (4.2 to 12.4)	-0.81 (-4.91 to 3.29)	0.700
STAI*					
	T2 (35w GA)	75.6 (67.3 to 83.9)	65.3 (58.0 to 72.6)	-9.88 (-19.44 to -0.32)	0.043
	T3 (1w a. d.)	67.5 (59.3 to 75.8)	63.6 (56.5 to 70.8)	-3.44 (-13.00 to 6.13)	0.481
	T4 (90d a. d.)	65.4 (56.3 to 74.4)	63.5 (54.9 to 72.1)	-1.44 (-11.00 to 8.12)	0.768
	T5 (6m a. d.)	64.8 (52.8 to 76.9)	62.6 (52.8 to 72.4)	-1.80 (-11.36 to 7.77)	0.713
State AI*					
	T2 (35w GA)	40.1 (36.0 to 44.2)	33.9 (30.3 to 37.6)	-6.55 (-11.75 to -1.35)	0.014
	T3 (1w a. d.)	35.1 (30.5 to 39.6)	32.2 (29.0 to 35.3)	-3.34 (-8.54 to 1.86)	0.209
	T4 (90d a. d.)	34.0 (29.2 to 38.8)	32.1 (27.9 to 36.2)	-2.36 (-7.56 to 2.84)	0.373
	T5 (6m a. d.)	33.5 (27.1 to 39.8)	32.1 (26.6 to 37.5)	-1.83 (-7.03 to 3.37)	0.491
Trait AI*					
	T2 (35w GA)	35.5 (31.2 to 39.9)	31.3 (27.0 to 35.6)	-2.93 (-7.87 to 2.01)	0.245
	T3 (1w a. d.)	32.5 (28.0 to 36.9)	31.5 (26.9 to 36.1)	0.30 (-4.64 to 5.24)	0.905
	T4 (90d a. d.)	31.4 (26.9 to 35.8)	31.4 (26.8 to 36.1)	1.33 (-3.61 to 6.27)	0.599
	T5 (6m a. d.)	31.4 (25.5 to 37.3)	30.5 (25.9 to 35.2)	0.43 (-4.51 to 5.37)	0.864
PTSD*					
	T2 (35w GA)	8.2 (4.1 to 12.2)	6.2 (3.0 to 9.4)	-1.11 (-4.53 to 2.31)	0.525
	T3 (1w a. d.)	7.1 (2.6 to 11.5)	7.6 (3.7 to 11.5)	1.33 (-2.09 to 4.76)	0.445
	T4 (90d a. d.)	4.6 (0.6 to 8.6)	5.4 (2.6 to 8.2)	1.64 (-1.79 to 5.06)	0.348
	T5 (6m a. d.)	5.5 (0.5 to 10.6)	5.0 (2.2 to 7.8)	0.29 (-3.13 to 3.72)	0.867

Table S5. Outcomes per protocol set for parenting stress and self-efficacy for mothers

Outcome		Mother<br>*Crude mean control*	*Crude mean intervention*	*Adjusted mean diff. (95%-CI)*	*p-value*
PSI-SF score*					
	T2 (35w GA)	71.1 (60.6 to 81.5)	63.4 (56.3 to 70.5)	-8.62 (-20.98 to 3.73)	0.171
	T3 (1w a. d.)	67.1 (57.8 to 76.3)	66.5 (59.6 to 73.4)	1.30 (-10.99 to 13.59)	0.836
	T4 (90d a. d.)	62.0 (53.0 to 71.0)	62.4 (55.6 to 69.2)	-3.52 (-19.44 to 12.39)	0.664
	T5 (6m a. d.)	67.5 (55.7 to 79.2)	62.1 (55.8 to 68.4)	-6.25 (-24.95 to 12.45)	0.513
Parental distress*					
	T2 (35w GA)	26.3 (20.6 to 31.9)	21.2 (18.0 to 24.3)	-3.22 (-7.29 to 0.85)	0.121
	T3 (1w a. d.)	23.9 (18.4 to 29.3)	22.2 (18.9 to 25.5)	0.08 (-4.69 to 4.86)	0.973
	T4 (90d a. d.)	21.6 (17.1 to 26.1)	20.3 (16.9 to 23.7)	0.54 (-4.03 to 5.11)	0.817
	T5 (6m a. d.)	23.4 (18.0 to 28.8)	21.6 (17.9 to 25.4)	0.12 (-5.79 to 6.03)	0.968
Parent-child dys. int.*					
	T2 (35w GA)	20.7 (17.1 to 24.3)	19.0 (16.7 to 21.4)	-0.64 (-4.16 to 2.88)	0.722
	T3 (1w a. d.)	19.1 (16.1 to 22.0)	19.6 (17.6 to 21.6)	1.27 (-1.62 to 4.15)	0.389
	T4 (90d a. d.)	18.0 (15.7 to 20.3)	18.4 (17.0 to 19.7)	0.06 (-3.18 to 3.29)	0.973
	T5 (6m a. d.)	19.3 (15.9 to 22.7)	17.8 (16.4 to 19.1)	-1.04 (-5.25 to 3.17)	0.628
Difficult child*					
	T2 (35w GA)	25.1 (20.8 to 29.4)	23.1 (20.3 to 26.0)	-2.10 (-7.35 to 3.14)	0.432
	T3 (1w a. d.)	24.1 (20.7 to 27.5)	24.7 (22.0 to 27.4)	0.88 (-3.41 to 5.16)	0.688
	T4 (90d a. d.)	23.3 (19.3 to 27.2)	23.8 (20.9 to 26.6)	-0.36 (-6.90 to 6.18)	0.915
	T5 (6m a. d.)	24.8 (20.3 to 29.3)	22.7 (20.1 to 25.2)	-3.44 (-10.64 to 3.77)	0.350
TOPSE score*					
	T2 (35w GA)	243.2 (224.0 to 262.4)	257.9 (246.3 to 269.5)	6.32 (-7.48 to 20.12)	0.369
	T3 (1w a. d.)	251.5 (229.9 to 273.0)	259.7 (247.8 to 271.6)	0.24 (-16.22 to 16.70)	0.977
	T4 (90d a. d.)	257.9 (239.3 to 276.6)	259.1 (247.7 to 270.5)	-6.60 (-22.47 to 9.26)	0.415
	T5 (6m a. d.)	256.3 (236.8 to 275.8)	262.7 (251.5 to 273.9)	-0.84 (-18.64 to 16.95)	0.926
emotion&affection*					
	T2 (35w GA)	51.0 (47.2 to 54.8)	54.7 (52.8 to 56.6)	3.68 (0.45 to 6.91)	0.025
	T3 (1w a. d.)	52.9 (49.6 to 56.2)	54.4 (52.2 to 56.5)	1.43 (-1.92 to 4.78)	0.403
	T4 (90d a. d.)	54.5 (51.8 to 57.3)	54.5 (52.1 to 56.8)	-0.08 (-3.36 to 3.20)	0.961
	T5 (6m a. d.)	55.5 (53.3 to 57.6)	55.8 (54.2 to 57.5)	0.35 (-1.72 to 2.42)	0.741
empathy&understanding*					
	T2 (35w GA)	49.6 (44.6 to 54.6)	50.5 (47.7 to 53.2)	1.08 (-2.90 to 5.06)	0.596
	T3 (1w a. d.)	49.7 (44.5 to 54.8)	50.7 (47.6 to 53.9)	1.28 (-2.86 to 5.42)	0.544
	T4 (90d a. d.)	52.7 (49.4 to 56.1)	51.8 (49.0 to 54.6)	-0.69 (-4.22 to 2.83)	0.700
	T5 (6m a. d.)	51.3 (46.3 to 56.3)	51.8 (49.3 to 54.4)	0.77 (-4.08 to 5.63)	0.755
pressures*					
	T2 (35w GA)	42.7 (35.4 to 50.0)	46.5 (41.0 to 52.0)	1.53 (-4.31 to 7.38)	0.607
	T3 (1w a. d.)	46.5 (39.6 to 53.5)	48.1 (42.5 to 53.7)	-0.74 (-7.30 to 5.82)	0.825
	T4 (90d a. d.)	46.6 (39.1 to 54.1)	45.5 (40.1 to 51.0)	-3.35 (-8.87 to 2.16)	0.233
	T5 (6m a. d.)	44.1 (35.9 to 52.4)	46.9 (41.2 to 52.6)	0.48 (-6.86 to 7.81)	0.899
self-acceptance*					
	T2 (35w GA)	45.7 (40.1 to 51.3)	52.5 (49.9 to 55.0)	4.32 (0.75 to 7.89)	0.018
	T3 (1w a. d.)	48.8 (42.7 to 54.9)	52.9 (50.1 to 55.7)	1.64 (-2.08 to 5.35)	0.387
	T4 (90d a. d.)	50.2 (44.5 to 55.9)	53.6 (50.7 to 56.5)	0.97 (-2.24 to 4.17)	0.555
	T5 (6m a. d.)	51.2 (46.1 to 56.3)	54.2 (52.0 to 56.4)	0.56 (-1.78 to 2.90)	0.640
learning&knowledge*					
	T2 (35w GA)	54.3 (50.9 to 57.6)	53.8 (52.0 to 55.6)	-1.59 (-4.26 to 1.07)	0.241
	T3 (1w a. d.)	53.5 (50.3 to 56.8)	53.6 (51.9 to 55.4)	-1.04 (-3.67 to 1.59)	0.437
	T4 (90d a. d.)	53.9 (50.5 to 57.2)	53.6 (51.7 to 55.6)	-1.38 (-4.07 to 1.32)	0.318
	T5 (6m a. d.)	54.2 (51.6 to 56.8)	54.0 (51.7 to 56.2)	-1.39 (-4.11 to 1.33)	0.316

Table S6. Outcomes per protocol set for parenting stress and self-efficacy for fathers

Outcome		Father<br>*Crude mean control*	*Crude mean intervention*	*Adjusted mean diff. (95%-CI)*	*p-value*
PSI-SF score*					
	T2 (35w GA)	69.6 (56.5 to 82.6)	61.8 (54.9 to 68.7)	-9.23 (-19.09 to 0.63)	0.067
	T3 (1w a. d.)	66.2 (56.4 to 76.1)	64.6 (55.4 to 73.7)	-2.97 (-12.83 to 6.90)	0.556
	T4 (90d a. d.)	62.2 (54.1 to 70.2)	60.6 (54.1 to 67.2)	-0.04 (-9.90 to 9.83)	0.994
	T5 (6m a. d.)	60.8 (52.1 to 69.5)	60.0 (53.6 to 66.5)	-1.50 (-11.36 to 8.36)	0.766
Parental distress*					
	T2 (35w GA)	21.6 (17.1 to 26.0)	19.3 (16.4 to 22.2)	-1.70 (-5.29 to 1.90)	0.355
	T3 (1w a. d.)	21.7 (17.2 to 26.2)	19.8 (15.9 to 23.6)	-1.36 (-4.95 to 2.23)	0.458
	T4 (90d a. d.)	19.6 (15.6 to 23.7)	18.2 (15.5 to 20.8)	-0.86 (-4.45 to 2.73)	0.638
	T5 (6m a. d.)	20.8 (15.1 to 26.5)	18.5 (15.7 to 21.3)	-1.67 (-5.26 to 1.92)	0.362
Parent-child dys. int.*					
	T2 (35w GA)	20.7 (16.5 to 24.9)	18.8 (16.4 to 21.1)	-3.05 (-5.98 to -0.12)	0.042
	T3 (1w a. d.)	19.9 (16.9 to 23.0)	19.3 (17.1 to 21.6)	-1.38 (-4.31 to 1.56)	0.358
	T4 (90d a. d.)	18.4 (16.3 to 20.6)	18.0 (16.1 to 19.8)	-0.99 (-3.92 to 1.95)	0.510
	T5 (6m a. d.)	17.6 (15.8 to 19.5)	17.8 (16.2 to 19.4)	-0.81 (-3.75 to 2.12)	0.587
Difficult child*					
	T2 (35w GA)	25.1 (20.2 to 30.0)	23.9 (21.1 to 26.8)	-2.64 (-7.13 to 1.86)	0.251
	T3 (1w a. d.)	24.6 (21.6 to 27.6)	25.5 (21.8 to 29.1)	-0.27 (-4.77 to 4.22)	0.905
	T4 (90d a. d.)	23.4 (19.8 to 27.0)	24.5 (21.6 to 27.3)	0.44 (-4.05 to 4.94)	0.847
	T5 (6m a. d.)	22.4 (19.7 to 25.0)	23.7 (20.8 to 26.6)	0.82 (-3.68 to 5.32)	0.721
TOPSE score*					
	T2 (35w GA)	252.2 (232.3 to 272.2)	261.5 (251.8 to 271.2)	11.26 (-2.67 to 25.20)	0.113
	T3 (1w a. d.)	258.5 (239.5 to 277.4)	263.7 (253.3 to 274.0)	9.87 (-4.09 to 23.83)	0.166
	T4 (90d a. d.)	260.4 (246.7 to 274.0)	268.8 (259.2 to 278.3)	14.17 (0.41 to 27.92)	0.044
	T5 (6m a. d.)	260.9 (242.3 to 279.5)	269.6 (261.7 to 277.4)	15.75 (1.99 to 29.50)	0.025
emotion&affection*					
	T2 (35w GA)	50.6 (45.8 to 55.4)	52.6 (50.8 to 54.3)	1.90 (-1.44 to 5.24)	0.264
	T3 (1w a. d.)	51.6 (47.1 to 56.2)	54.0 (52.2 to 55.7)	2.28 (-1.06 to 5.62)	0.180
	T4 (90d a. d.)	53.6 (51.3 to 56.0)	54.6 (52.2 to 57.0)	0.95 (-2.39 to 4.29)	0.578
	T5 (6m a. d.)	53.3 (50.1 to 56.5)	54.7 (53.1 to 56.4)	1.40 (-1.94 to 4.74)	0.411
empathy&understanding*					
	T2 (35w GA)	47.9 (40.8 to 55.1)	49.9 (46.7 to 53.0)	2.90 (-2.03 to 7.82)	0.249
	T3 (1w a. d.)	47.6 (42.8 to 52.3)	51.8 (49.1 to 54.5)	4.85 (-0.08 to 9.78)	0.054
	T4 (90d a. d.)	50.6 (46.7 to 54.4)	51.2 (48.1 to 54.3)	0.85 (-4.08 to 5.77)	0.737
	T5 (6m a. d.)	51.0 (47.0 to 55.0)	52.5 (50.6 to 54.5)	2.26 (-2.67 to 7.18)	0.370
pressures*					
	T2 (35w GA)	50.2 (44.1 to 56.3)	51.4 (47.3 to 55.5)	2.34 (-2.52 to 7.19)	0.345
	T3 (1w a. d.)	49.8 (42.0 to 57.6)	51.4 (47.6 to 55.1)	2.77 (-2.09 to 7.62)	0.264
	T4 (90d a. d.)	53.1 (47.4 to 58.8)	54.8 (51.9 to 57.6)	2.86 (-1.99 to 7.72)	0.248
	T5 (6m a. d.)	50.9 (43.2 to 58.6)	53.5 (50.6 to 56.5)	3.84 (-1.02 to 8.69)	0.121
self-acceptance*					
	T2 (35w GA)	50.4 (44.7 to 56.1)	55.0 (52.6 to 57.5)	5.51 (1.69 to 9.32)	0.005
	T3 (1w a. d.)	51.5 (45.9 to 57.1)	54.1 (51.6 to 56.7)	3.53 (-0.28 to 7.34)	0.069
	T4 (90d a. d.)	52.1 (48.2 to 55.9)	55.2 (53.2 to 57.2)	4.01 (0.19 to 7.82)	0.039
	T5 (6m a. d.)	52.9 (47.7 to 58.0)	55.5 (53.5 to 57.4)	3.51 (-0.31 to 7.32)	0.071
learning&knowledge*					
	T2 (35w GA)	50.6 (46.6 to 54.5)	52.6 (50.2 to 55.1)	2.90 (-1.28 to 7.08)	0.174
	T3 (1w a. d.)	50.2 (45.3 to 55.1)	52.4 (50.0 to 54.8)	3.02 (-1.16 to 7.20)	0.157
	T4 (90d a. d.)	51.0 (46.6 to 55.4)	53.0 (49.2 to 56.8)	2.85 (-1.33 to 7.03)	0.181
	T5 (6m a. d.)	52.9 (49.8 to 55.9)	53.3 (50.8 to 55.8)	1.33 (-2.85 to 5.51)	0.534

Table S7. Intraclass correlations (ICC) for mothers and fathers for all scores between all timepoints

Outcome	Parent	ICC consistency
CES-D		
	mother	0.893
	father/other caregiver	0.738
STAI*		
	mother	0.921
	father/other caregiver	0.871
State AI*		
	mother	0.887
	father/other caregiver	0.825
Trait AI*		
	mother	0.942
	father/other caregiver	0.901
PTSD*		
	mother	0.949
	father/other caregiver	0.894
PSI-SF score*		
	mother	0.814
	father/other caregiver	0.863
Parental distress*		
	mother	0.900
	father/other caregiver	0.907
Parent-child dys. int.		
	mother	0.711
	father/other caregiver	0.807
Difficult child*		
	mother	0.805
	father/other caregiver	0.805
TOPSE score*		
	mother	0.944
	father/other caregiver	0.837
emotion&affection*		
	mother	0.771
	father/other caregiver	0.676
empathy&understanding*		
	mother	0.875
	father/other caregiver	0.647
pressures*		
	mother	0.945
	father/other caregiver	0.884
self-acceptance*		
	mother	0.950
	father/other caregiver	0.854
learning&knowledge*		
	mother	0.842
	father/other caregiver	0.733

Table S8. Pearson between fathers and mothers for all scores all timepoints combined

Outcome	Pearson pairwise correlation
CES-D	0.443
STAI*	0.395
State AI*	0.361
Trait AI*	0.404
PTSD*	0.198
PSI-SF score*	0.324
Parental distress*	0.310
Parent-child dys. int.	0.365
Difficult child*	0.425
TOPSE score*	0.364
emotion&affection*	0.333
empathy&understanding*	0.400
pressures*	0.230
self-acceptance*	0.357
learning&knowledge*	0.296

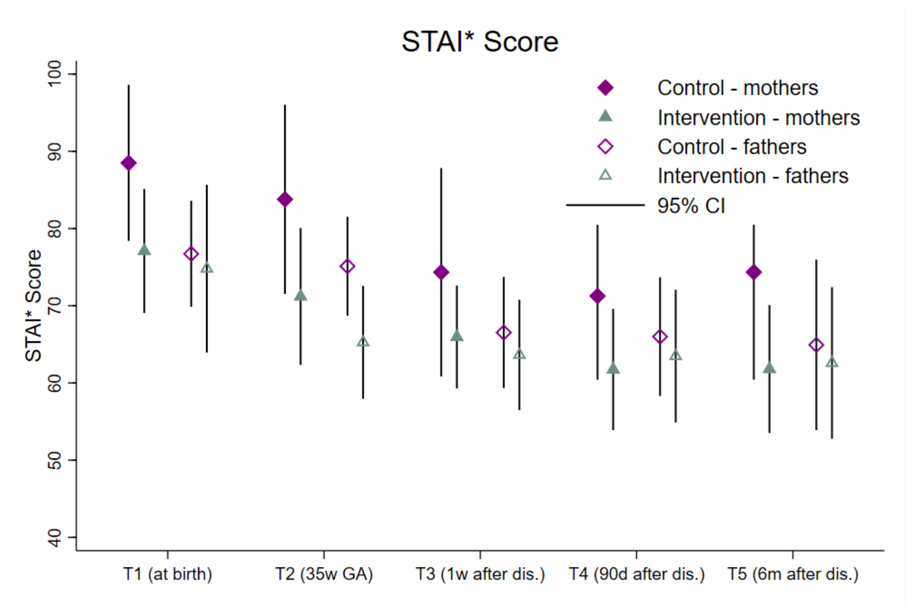

Figure S1. Course of anxiety (STAI) scores from birth until six months after preterm infants' discharge in mothers and fathers of the CG and the IG.

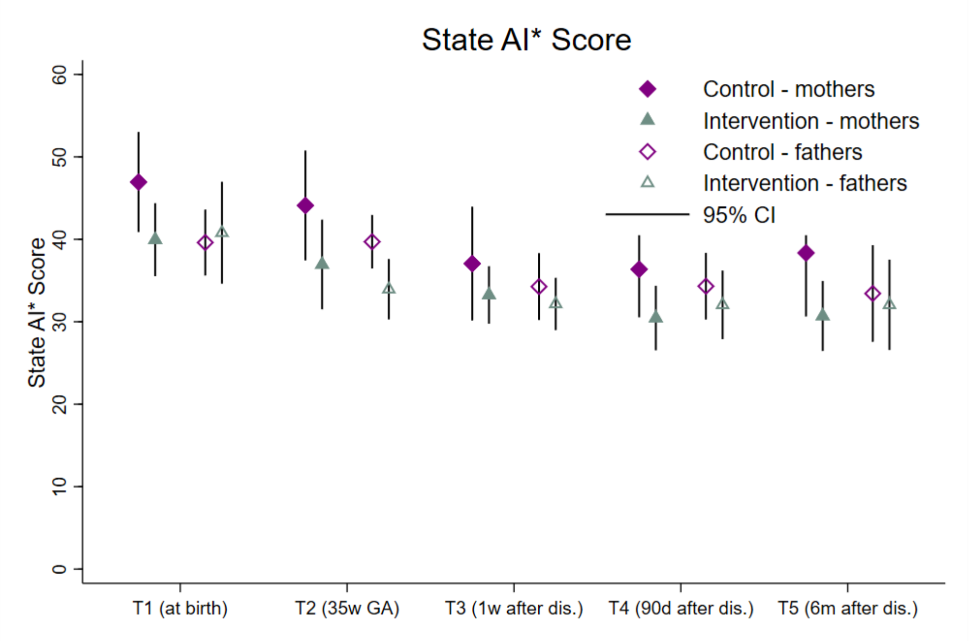

Figure S2. Course of state anxiety scores from birth until six months after preterm infants’ discharge in mothers and fathers of the CG and the IG.

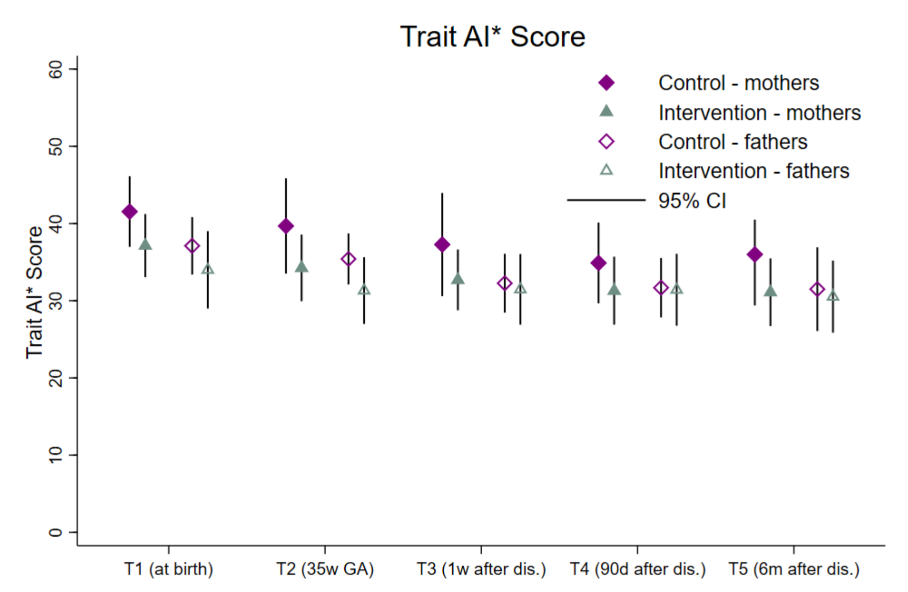

Figure S3. Course of trait anxiety scores from birth until six months after preterm infants’ discharge in mothers and fathers of the CG and the IG.

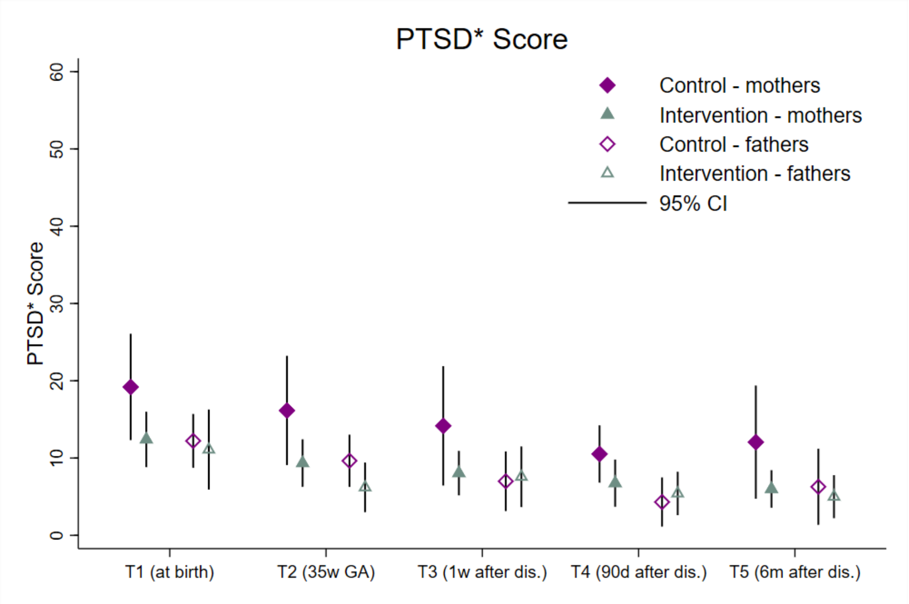

Figure S4. Course of posttraumatic stress disorders (PTSD) scores from birth until six months after preterm infants' discharge in mothers and fathers of the CG and the IG.
